# Allelic Variation in *CXCL16* Determines CD3^+^ T Lymphocyte Susceptibility to Equine Arteritis Virus Infection and Establishment of Long-Term Carrier State in the Stallion

**DOI:** 10.1371/journal.pgen.1006467

**Published:** 2016-12-08

**Authors:** Sanjay Sarkar, Ernest Bailey, Yun Young Go, R. Frank Cook, Ted Kalbfleisch, John Eberth, R. Lakshman Chelvarajan, Kathleen M. Shuck, Sergey Artiushin, Peter J. Timoney, Udeni B. R. Balasuriya

**Affiliations:** 1 Maxwell H. Gluck Equine Research Center, Department of Veterinary Science, University of Kentucky, Lexington, Kentucky, United States of America; 2 Department of Biochemistry and Molecular Genetics, School of Medicine, University of Louisville, Louisville, Kentucky, United States of America; University of Bern, SWITZERLAND

## Abstract

Equine arteritis virus (EAV) is the causative agent of equine viral arteritis (EVA), a respiratory, systemic, and reproductive disease of horses and other equid species. Following natural infection, 10–70% of the infected stallions can become persistently infected and continue to shed EAV in their semen for periods ranging from several months to life. Recently, we reported that some stallions possess a subpopulation(s) of CD3^+^ T lymphocytes that are susceptible to *in vitro* EAV infection and that this phenotypic trait is associated with long-term carrier status following exposure to the virus. In contrast, stallions not possessing the CD3^+^ T lymphocyte susceptible phenotype are at less risk of becoming long-term virus carriers. A genome wide association study (GWAS) using the Illumina Equine SNP50 chip revealed that the ability of EAV to infect CD3^+^ T lymphocytes and establish long-term carrier status in stallions correlated with a region within equine chromosome 11. Here we identified the gene and mutations responsible for these phenotypes. Specifically, the work implicated three allelic variants of the equine orthologue of *CXCL16* (*EqCXCL16*) that differ by four non-synonymous nucleotide substitutions (XM_00154756; c.715 A → T, c.801 G → C, c.804 T → A/G, c.810 G → A) within exon 1. This resulted in four amino acid changes with EqCXCL16S (XP_001504806.1) having Phe, His, Ile and Lys as compared to EqCXL16R having Tyr, Asp, Phe, and Glu at 40, 49, 50, and 52, respectively. Two alleles (*EqCXCL16Sa*, *EqCXCL16Sb*) encoded identical protein products that correlated strongly with long-term EAV persistence in stallions (P<0.000001) and are required for *in vitro* CD3^+^ T lymphocyte susceptibility to EAV infection. The third (*EqCXCL16R*) was associated with *in vitro* CD3^+^ T lymphocyte resistance to EAV infection and a significantly lower probability for establishment of the long-term carrier state (viral persistence) in the male reproductive tract. *EqCXCL16Sa* and *EqCXCL16Sb* exert a dominant mode of inheritance. Most importantly, the protein isoform EqCXCL16S but not EqCXCL16R can function as an EAV cellular receptor. Although both molecules have equal chemoattractant potential, EqCXCL16S has significantly higher scavenger receptor and adhesion properties compared to EqCXCL16R.

## Author Summary

A variable proportion of EAV infected stallions (10–70%) may become persistently infected and continuously shed the virus exclusively in their semen after recovery from acute infection. Previous studies in our laboratory have shown that stallions with the CD3^+^ T lymphocyte susceptibility phenotype to *in vitro* EAV infection are at higher risk of becoming persistently infected carriers compared to those that lack this phenotype. Here genetic and experimental studies were used to demonstrate that *CXCL16* in the horse codes for two proteins, one associated with resistance and the other associated with susceptibility of CD3^+^ T lymphocytes to EAV infection. The two proteins are the result of four nucleotide substitutions in exon 1 of the equine *CXCL16* gene. These alleles determine the outcome of *in vitro* infection of CD3^+^ T lymphocytes with EAV and are strongly associated with the establishment and maintenance of long-term carrier state in stallions. *In vitro* studies demonstrated that one form of CXCL16 protein (CXCL16S) is one of the cellular receptors for EAV and has higher scavenger activity and adhesion ability as compared to the form of the protein associated with resistance (CXCL16R).

## Introduction

Equine arteritis virus (EAV) is a single-stranded, positive-sense RNA virus that belongs to the family *Arteriviridae* in the order *Nidovirales* [1–3]. It is the causative agent of equine viral arteritis (EVA) a respiratory, systemic, and reproductive disease of horses [2, 4, 5]. While most naturally acquired EAV infections are clinically inapparent, relatively virulent field strains of EAV periodically emerge around the world giving rise to outbreaks of EVA [6, 7]. The disease is characterized by fever (greater than 41°C); depression; leukopenia; rhinitis often accompanied by nasal discharge; urticaria; and edema [8]. Abortion is a frequent outcome in naïve pregnant mares and congenital infection in neonatal foals is characterized by severe, fulminating interstitial pneumonia [9]. In the stallion, EAV is shed in semen during the acute phase of the infection and in some individuals, for a short time during the convalescent period until they clear the virus entirely from all body tissues [10]. However, in contrast, EAV establishes long-term persistent infection in 10–70% of infected stallions and these constantly shed virus in their semen for extended periods (years or even life long) [8, 11, 12]. The mechanism of long-term persistence of EAV in the reproductive tract of stallions is not well understood. It has been shown that EAV persistence in sexually intact post-pubertal colts or stallions is testosterone dependent [13, 14]. Persistently infected stallions play an important role in maintenance and perpetuation of the virus in equine populations by transmitting the virus during breeding to naïve susceptible mares and can be responsible for outbreaks of EVA [8, 13–17]. The use of virus-infective frozen or chilled semen for artificial insemination and embryo transfer can increase the risk of spread of EAV [18].

In previous studies in our laboratory, it has been shown that the experimentally derived virulent Bucyrus strain (VBS) of EAV can infect CD3^+^ T lymphocytes *in vitro* from some but not all horses [19]. In one study, 310 horses of Thoroughbred, Standardbred, Saddlebred, and Quarter horse breeds were tested and their phenotypes identified with respect to *in vitro* infection of their CD3^+^ T lymphocytes by the VBS of EAV [20]. Those whose CD3^+^ T lymphocytes could be infected *in vitro* with this virus strain were identified as susceptible, and those whose cells were not infected were identified as resistant [20]. A genome wide association study (GWAS) identified a common haplotype associated with the *in vitro* CD3^+^ T lymphocyte susceptible phenotype in a region of equine chromosome 11 (ECA11:49572804–49643932) whose distribution was consistent with a dominant mode of inheritance [20]. Subsequently, it has also been demonstrated that stallions with the CD3^+^ T lymphocyte susceptibility phenotype to *in vitro* EAV infection are also at a significantly higher risk of becoming persistently infected carriers compared to those that lack this phenotype [21].

The primary objective of this study was to identify the specific gene(s) involved in the *in vitro* CD3^+^ T lymphocyte susceptibility to EAV infection and also the molecular mechanism(s) responsible for this phenomenon. We have combined contemporary genomics, molecular biology, and proteomics techniques to demonstrate that a specific gene in equine chromosome 11 (ECA11), equine *CXCL16* (*EqCXCL16*), plays an essential role in the *in vitro* CD3^+^ T lymphocyte susceptibility to EAV infection. In this study, we have shown the *EqCXCL16* gene has three alleles, two coding for the susceptibility phenotype (*EqCXCL16Sa* and *EqCXCL16Sb*) and one coding for the resistant phenotype (*EqCXCL16R*). Furthermore, compelling evidence is provided that allelic variation within *EqCXCL16* is a major determining factor for establishment of long-term persistent EAV infection in the stallion reproductive tract. This study also identified key functional differences between the EqCXCL16S and EqCXCL16R proteins, highlighting the biological significance of mutations present in the *EqCXCL16* gene.

## Results

### Non-synonymous nucleotide sequence variation within the 48M-51M region of ECA11 (SNP Discovery)

Three horses were selected for whole genome sequence analysis to identify nucleotide substitutions within the 3 megabase target region of ECA11 (ECA11: 48M-51M) associated with *in vitro* susceptibility or resistance of CD3^+^ T lymphocytes to infection with EAV. Two horses (Thoroughbred-10 [TB10] and Standardbred-22 [STB22]) possessed CD3^+^ T lymphocytes with the EAV susceptible phenotype while the corresponding cells in Thoroughbred-3 (TB03) were resistant. Sequencing of 500 bp paired end libraries was accomplished using an Illumina HiSeq2000 platform to a depth of approximately 30× coverage. Following alignment to the reference genome (Ecab 2.0), the distribution of SNPs within ECA11: 48M-51M was compared to identify differences between susceptible and resistant horses. SNPs of interest were those which occurred in both susceptible horses but not the resistant horse. The analysis focused on identification of SNPs causing non-synonymous mutations within exons for genes annotated by Ensembl as these were considered to have a high likelihood of influencing the phenotype. In total, 12 non-synonymous SNPs were found in exons for eight annotated genes within the target region (Table 1). No translational frame-shift insertions or deletions (indels) were found within any previously annotated exon.

**Table 1:** Non-synonymous nucleotide changes in eight genes located in the 48M-51M region of ECA11.

rs Number	Genomic Coordinates	^1^Gene	SNP Change^2^	aa Change^2^
rs395376880	48640104	WSCD1	T→C	Trp→Arg
rs869312026	49070071	NLRP1	T→C	Ser→Pro
rs68886110	49084976	NLRP1	T→C	Trp→Arg
rs395626725	49427602	ZNF (1425)	A→G	Glu→Gly
rs396124728	49438260	ZNF (14599)	A→G	Ile→Val
rs394122974	49457063	ZNF (1660)	A→G	Met→Val
rs782829411	49746951	CXCL16	A→T	Tyr→Phe
rs782894239	49746977	CXCL16	G→C	Asp→His
rs782894239	49746980	CXCL16	T→A	Phe→Ile
rs782838921	49746986	CXCL16	G→A	Glu→Lys
rs869312027	50591820	SHBG	C→A	Pro→Gln
rs68875925	50838580	KCNAB3	A→G	Ile→Val

^1^WSCD1—WSC Domain containing 1; NLRP1—NLR family pyrin domain containing 1; ZNF—Zinc finger protein; CXCL16—C-X-C motif chemokine ligand 16; SHBG—sex hormone binding globulin; and KCNAB3—potassium voltage-gated channel, shaker-related subfamily, beta member 3.

^2^The SNP and amino acid residue changes are identified with reference to Ensembl reference sequences in S2 Table.

### SNPs within the equine orthologue of *CXCL16* (*EqCXCL16*) are associated with susceptibility of CD3^+^ T lymphocytes to EAV infection

Potential associations between SNPs identified by genomic sequencing and the CD3^+^ T lymphocyte EAV susceptibility phenotype were determined by sequencing PCR amplified fragments spanning each of the 12 SNP locations from a subset of the 240 horses (resistant [n = 2] and susceptible [n = 8]) (Table 2). Only the SNPs associated with the equine orthologue of the gene encoding the chemokine *CXCL16* (*EqCXCL16*) showed a complete association; the two resistant horses were homozygous for the A, G, T, and G SNPs at (XM_00154756) c.715, c.801, c.804, and c.810, while all eight susceptible horses were either homozygous for the SNPs T, C, A, and A or were heterozygous A/T, G/C, T/A, and G/A at those positions, respectively_,_ This observation is consistent with genetic dominance for the EAV CD3^+^ T lymphocyte susceptibility phenotype as reported previously [20].

**Table 2:** Distribution of non-synonymous SNPs for eight candidate genes among ten horses with resistant (two) or susceptible (eight) phenotypes for *in vitro* infection of CD3^+^ T lymphocytes with EAV VBS.

^1^Gene/ SNP	SNP Genotype	Number of Susceptible Horses	Number of Resistant Horses
NLRP1 **rs869312026**	CC	1	2
	CT	5	0
	TT	2	0
NLRP1 **rs68886110**	CC	2	0
	CT	5	1
	TT	1	1
WSDC1 **rs395376880**	AA	3	2
	AG	2	0
	GG	3	0
ZNF1425 **rs395626725**	AA	4	1
	AG	3	0
	GG	1	1
ZNF14599 **rs396124728**	AA	0	1
	AG	3	1
	GG	5	0
ZNF1660 **rs394122974**	AA	1	1
	AG	3	0
	GG	4	1
KCNBP **rs68875925**	AA	1	0
	AG	4	1
	GG	3	1
SHBG **rs869312027**	AA	3	0
	AC	4	2
	CC	0	0
CXCL16; **rs782829411**	AA	0	2
	AT	6	0
	TT	2	0
CXCL16 **rs782894239**	GG	0	2
	GC	6	0
	CC	2	0
CXCL16 **rs782894239**	TT	0	2
	AT	6	0
	AA	2	0
CXCL16 **rs782838921**	GG	0	2
	AG	6	0
	AA	2	0

^1^WSCD1—WSC Domain containing 1; NLRP1—NLR family pyrin domain containing 1; ZNF—Zinc finger protein; CXCL16—C-X-C motif chemokine ligand 16; SHBG—sex hormone binding globulin; and KCNAB3—potassium voltage-gated channel, shaker-related subfamily, beta member 3.

### *EqCXCL16* alleles and predicted protein products for four horse breeds

To investigate the distribution of the four SNPs described above in a larger, more representative sample of the horse population, exon 1 of *EqCXCL16* was PCR amplified and sequenced from 240 horses (Thoroughbred [n = 67], Standardbred [n = 60], Quarter Horse [n = 53], and Saddlebred horses [n = 60]; see S1 Table for primer sequences). The 240 horses had previously been characterized as EAV CD3^+^ T resistant (n = 85) or susceptible (n = 155). Based on the annotation for the equine reference *CXCL16* (XM_001504756) sequence, all resistant horses (n = 85) were homozygous for the SNPs A, G, T, and G at positions 715, 801, 804, and 810. However, all susceptible horses were homozygous or heterozygous at those positions and possessed the SNPs T, C, A, A or T, C, G, A (n = 154) at the respective positions. A single (n = 1) homozygote was observed for a putative haplotype possessing the combination T, C, G, A; that individual was phenotyped as susceptible. These two susceptibility alleles (T, C, A, A and T, C, G, A) were designated *EqCXCL16Sa* and *EqCXCL16Sb*, respectively. The predicted amino acid sequences were identical for *EqCXCL16Sa* and *EqCXCL16Sb* as described below. The allele for resistance was recessive and was characterized by A, G, T, and G at positions 715, 801, 804, and 810. This allele was designated *EqCXCL16R* and was identical to the reference genome sequence (Ecab 2.0). No additional alleles were found in exon 1 of *EqCXCL16* among 47 horses of 20 other breeds tested during the study. The gene frequencies of the three *CXCL16* alleles are shown in Table 3 for Thoroughbred, Quarter Horse, Saddlebred, and Standardbred horse breeds.

**Table 3:** Gene frequencies among Thoroughbred (n = 67), Quarter Horse (n = 53), Saddlebred (n = 60), and Standardbred (n = 60) horses for alleles *CXCL16R*, *CXCL16Sa*, and *CXCL16Sb*.

**Breed**	*CXCL16R*	*CXCL16Sa*	*CXCL16Sb*
**Thoroughbred**	0.882	0.078	0.040
**Quarter Horse**	0.735	0.255	0.010
**Saddlebred**	0.344	0.500	0.156
**Standardbred**	0.386	0.596	0.018

### Predicted amino acid sequences encoded by *EqCXCL16Sa*, *EqCXCL16Sb*, and *EqCXCL16R*; three alleles and two proteins

The originally identified SNPs were non-synonymous mutations, predicting a change of amino acids in the protein (Table 1). The predicted amino acid sequences for this region of exon 1 specify phenylalanine (Phe), histidine (His), isoleucine (Ile) and lysine (Lys) at amino acid positions 40, 49, 50, and 52 respectively, for both EqCXCL16Sa and EqCXCL16Sb based on the horse reference sequence, XM_001504756. The predicted amino acids at those positions for *EqCXCL16R* were tyrosine (Tyr), aspartic acid (Asp), phenylalanine (Phe), and glutamic acid (Glu). These are non-conservative substitutions.

### Association of the *EqCXCL16* genotypes with the phenotypes for CD3^+^ T lymphocytes susceptibility

The distribution of the two predicted EqCXCL16 proteins among the 240 Thoroughbred, Quarter Horses, Saddlebred, and Standardbred was compared to the distribution of their CD3^+^ T lymphocyte phenotypes for resistance or susceptibility to equine arteritis virus infection. As can be seen in Table 4, the association was complete. This observation is particularly noteworthy since it occurred across a group including 4 different horse breeds.

**Table 4:** Association of *CXCL16* alleles with susceptibility and resistance of CD3^+^ T lymphocytes to EAV infection (n = 240).

*CXCL16* alleles	Susceptible	Resistant
*EqCXCL16R/EqCXCL16R*	0	85
EqCXCL16S*/ EqCXCX16 (S or R)	155	0

*CXCL16S represents both alleles *CXCL16Sa* and *CXCL16Sb*. P<0.0001 using Fisher's Exact Test.

The 5 carriers with the resistance genotype came from 5 different breeds (Saddlebred, Friesian, Lusitano, Thoroughbred, and Quarter Horse) while the 11 non-carriers with susceptibility genotypes came from 8 different breeds (Belgian Warmblood, Belgian draft, Saddlebred, Standardbred (4), Paint, Friesian, Hanoverian, and Quarter Horse.

### Inter-species comparison of predicted amino acid sequences within exon 1 of *CXCL16*

**Fig 1:**
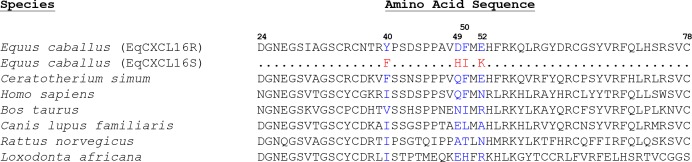
Alignment of predicted amino acid sequences within exon 1 of CXCL16 proteins from seven different mammalian species. The *Equus caballus* CXCL16R protein (amino acid residues 24–78) is depicted at the top, followed by *Equus caballus* CXCL16S protein and CXCL16 of other mammalian species.

The predicted amino acid sequences within exon 1 of *CXCL16* was compared for seven species using the reference genome sequences from white rhinoceros (*Ceratotherium simum*), horse (*Equus caballus*), dog (*Canis lupus familiaris* or *Canis familiaris*), human (*Homo sapiens*), domestic rat (*Rattus norvegicus*), cattle (*Bos taurus*), and African elephant (*Loxodonta africana*) (Fig 1). As noted above, the horse genome reference sequence (Ecab 2.0) is identical to *EqCXCL16R* (Fig 1). Although the sequences depicted in Fig 1 have some shared elements such as a GN-GS motif (positions 2–6 Fig 1) or a C residue at position 11, this region is not highly conserved between species and different amino acid side chains are permitted at sites equivalent to those involving polymorphism within CXCL16 in the horse. However, position 40 of EqCXCL16R differs in that Tyr is hydrophilic; while in EqCXCL16S and all other species examined, this site is occupied by a non-polar residue. In addition, EqCXCL16S differs at position 49 in that His has a basic side chain; whereas in the case of EqCXCL16R, along with other species, this position is generally occupied by amino acids with acidic or polar side chains (Fig 1). In contrast, the identities of amino acids or amino acid side chain properties at positions 50/52 in EqCXCL16S and EqCXCL16R are observed in other species, with Phe in EqCXCL16R seen in humans along with another perissodactyl, the white rhinoceros, and Ile is present in cattle. Similarly, at position 52, Glu in EqCXCL16R is also present in the white rhinoceros; although Lys in EqCXCL16S is not found in any of the other species shown in Fig 1, positively charged Arg is observed in cattle and African elephants. These results suggest that exon 1 of *CXCL16* has been exposed to considerable selective pressure over time and imply adaptive evolution for this region unique to each mammalian species.

### Establishment of association of *CXCL16* haplotypes with long-term carrier status in stallions

We previously reported an association between susceptibility of CD3^+^ T lymphocytes to infection and the ability of EAV virus to establish the long-term carrier state in stallions (7). Following natural infection, some stallions continue to shed virus in the semen for long periods (more than one year to lifelong) after EAV is no longer present in blood and nasal secretions [2, 22] and as such become reservoirs for the maintenance of this virus in equid populations. Therefore, we analyzed semen from 77 stallions comprising 24 different breeds that had been infected with EAV and their status as long-term carrier animals determined (shedders; Table 5). Since the *CXCL16* alleles showed complete association with the CD3^+^ T lymphocyte phenotype across several breeds, data for the carrier-status phenotype were pooled for 77 stallions from different breeds. Of these, 37 were identified as long-term carriers (stallions EAV seropositive and shedding detectable levels of virus in semen more than one year after initial infection) while 40 were identified as non-carriers (stallions seropositive for EAV, but had apparently cleared the virus from the reproductive tract [non-shedders]) based on absence of detectable virus in semen post-infection. Exon 1 of *EqCXCL16* was PCR amplified and sequenced from all 77 stallions to determine each stallion’s genotype. The results demonstrated a strong, although incomplete, association between long-term EAV carrier status in stallions and the presence of at least one copy of the *EqCXCL16Sa* or *EqCXCL16Sb* allele among 86% of the stallions with that phenotype (Table 6). In contrast, 73% of stallions that had been infected with EAV but were negative for virus in their semen following infection were homozygous for *EqCXCL16R* (Table 6).

**Table 5:** Breeds of stallions and status as carriers and non-carriers for shedding of EAV in semen more than one year post EAV infection.

Breed	Carriers (n = 37)	Non-Carriers (n = 40)
Quarter Horse	7	7
Friesian	3	4
Rocky Mountain Saddle	1	0
Tennessee Walking Horse	3	0
American Standardbred	2	5
Paint	1	3
Selle Francais	1	0
Hanoverian	2	3
Belgian Warmblood	1	0
Swedish Warmblood	1	
Thoroughbred	5	2
Arabian	2	4
Lusitano	1	0
Andalusian	1	0
American Saddlebred	1	1
Dutch Warmblood	4	3
Westphalian	1	0
Holsteiner	0	1
Percheron	0	1
Belgian	0	1
Trakehner	0	1
Danish Warmblood	0	1
Zangersheide	0	1
Warmblood (pedigree unknown; owner designation)	0	2

**Table 6:** Association of *CXCL16* alleles with carrier and non-carrier status of stallions for EAV shedding in semen (n = 77).

*CXCL16* alleles	Carriers (Semen Positive)	Non-Carriers (Semen Negative)
*EqCXCL16R/EqCXCL16R*	5	29
EqCXCL16S*/ EqCXCX16 (S or R)	32	11

*CXCL16S represents both alleles *CXCL16Sa* and *CXCL16Sb*. P<0.000001 using Fisher's Exact Test without regard to breed.

### Effect of amino acid substitutions in the EqCXCL16 protein on response to EAV infection

**Fig 2:**
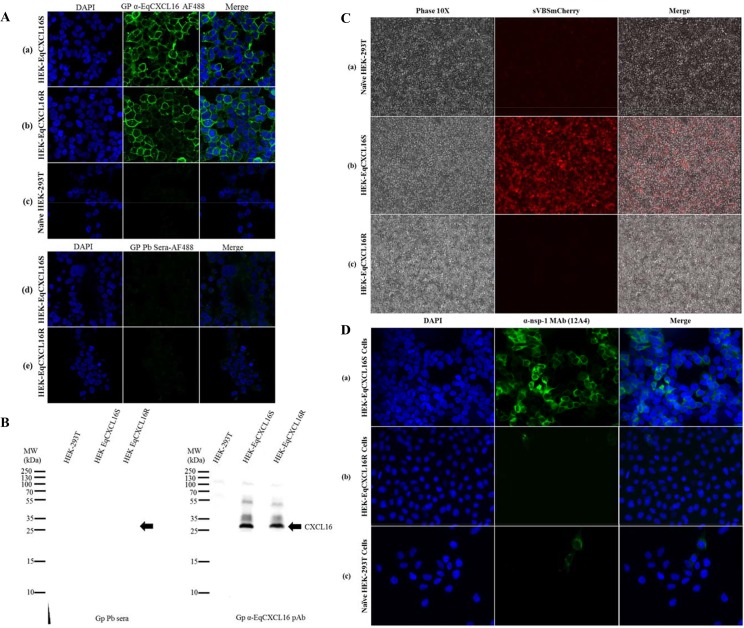
The ability of EqCXCL16 to function as an entry receptor for EAV is determined by amino acid substitutions in the N-terminal ectodomain of the protein encoded by exon 1. A) Stable HEK-293T cell lines were generated by transfection with the pJ609-EqCXCL16S or pJ609-EqCXCL16R plasmid DNA followed by selection with antibiotic puromycin. These cells (panel a: EqCXCL16S, panel b: EqCXCL16R) were surface stained with Gp α-EqCXCL16 Ab or guinea pig pre-bleed (Gp Pb) sera (panels d and e) as primary antibodies, followed by goat anti-Gp IgG(H+L) conjugated to Alexa Fluor 488 and analyzed by confocal microscopy to confirm the expression of EqCXCL16 protein. Naïve HEK-293T cells were also stained with Gp α-EqCXCL16 Ab (panel c). B) Naïve HEK-293T and stable HEK-EqCXCL16S and HEK-EqCXCL16R cells were lysed in RIPA cell-lysis buffer and an equal amount of lysates were analyzed by WB using Gp Pb sera or Gp α-EqCXCL16 Ab. Arrow indicates the absence or presence of EqCXCL16 protein in the WB membrane (molecular weight approximately 30 kDa). C) Naïve HEK-293T (a), HEK-EqCXCL16S (b), and HEK-EqCXCL16R (c) cells were infected with EAV sVBSmCherry (synthetic virus expressing mCherry) [24] at a multiplicity of infection of 1 (MOI = 1). At 12 hours post infection (hpi), cells were washed with PBS, fixed in 4% paraformaldehyde (PFA) and analyzed with an inverted immunofluorescence microscope for the expression of mCherry (red) as an indicator of EAV infection. Markedly increased numbers of stable HEK-EqCXCL16S cells were shown to express mCherry compared to naïve HEK-293T and stable HEK-EqCXCL16R cells. D) The role of EqCXCL16 variants on EAV infection and gene expression was analyzed in the EAV sVBSmCherry infected cells. a) HEK-EqCXCL16S, b) HEK-EqCXCL16R, and c) naïve HEK-293T cells were infected with EAV sVBSmCherry at an MOI = 1. After fixation in 4% PFA at 12 hpi, cells were analyzed with an inverted immunofluorescence microscope for the expression of EAV nsp-1 gene using a monoclonal antibody, α-nsp-1 MAb (12A4). Images are representative of three independent experiments.

Recently, we demonstrated that the membrane-associated form of the chemokine encoded by the *EqCXCL16Sa* allele can function as a host-cell receptor for EAV binding and entry [23]. Since the four predicted amino acid substitutions discovered in exon 1 of *EqCXCL16R* compared to *EqCXCL16Sa/b* appear to be non-conservative, we hypothesized they might modify the functional EAV receptor properties of the resultant EqCXCL16R molecule. Consequently, as described previously for EqCXCL16S [23], we established an HEK-293T cell line for stable expression of the transmembrane form of the EqCXCL16R protein. Stable expression of the respective proteins from HEK-EqCXCL16S and HEK-EqCXCL16R cells were compared using confocal microscopy (Fig 2A) and Western blot (WB) analysis (Fig 2B) using guinea pig (Gp) α-EqCXCL16 antisera. The results confirmed that the variant forms of EqCXCL16 were produced in apparently similar amounts and that they were both clearly associated with the plasma membrane (Fig 2A).

Infection of HEK-EqCXCL16S with the EAV sVBSmCherry encoding the mCherry fluorescent protein confirmed our previous reported findings that by 12 hpi the fluorophore was expressed in almost every cell (Fig 2C, panel b). In contrast, expression of mCherry in HEK-EqCXCL16R cells infected at the same MOI with EAV sVBSmCherry was almost undetectable at the same time point and as such was equivalent to that observed in mock transfected HEK-293T cells (Fig 2C, panels a and c). This result was confirmed in experiments where EAV gene expression following infection was detected by an indirect immunofluorescence assay (IFA) using a monoclonal antibody against viral nonstructural protein 1 (nsp-1). Although nsp-1 expression was detectable in a few EAV infected HEK-EqCXCL16R and mock transfected HEK-293T cells (Fig 2D, panels b and c), the numbers were markedly lower than observed in similarly infected HEK-EqCXCL16S cells (Fig 2D, panel a). These results demonstrate that the plasma membrane-associated EqCXCL16S isoform can function as an efficient cellular receptor for EAV while EqCXCL16R does not.

### EAV VBS binds directly to EqCXCL16S but not to EqCXCL16R *in vitro*

**Fig 3:**
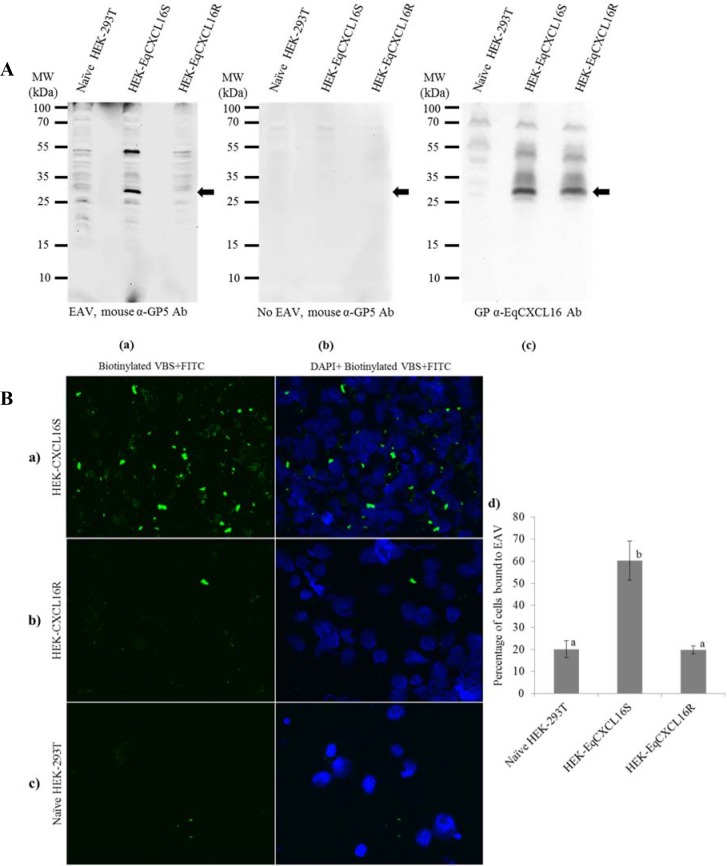
Binding of EAV to EqCXCL16 is determined by amino acid substitutions within the N-terminal ectodomain of the protein encoded by exon 1. A). Effect of amino acid differences between EqCXCL16S and EqCXCL16R on EAV binding as determined by a combination of VOPBA and Far-WB analysis. Naïve HEK-293T, stable HEK-EqCXCL16S and HEK-EqCXCL16R cells were lysed in RIPA buffer, the lysate proteins separated using 12% SDS-PAGE and transferred onto a PVDF membrane. Membrane bound proteins were denatured and renatured using sequentially decreasing concentrations of Gn-HCl following which the membranes were blocked and incubated either with purified EAV VBS (15 μg/ml) in protein binding buffer (a) or with protein binding buffer without purified EAV VBS (b). After washing, membranes were incubated with α-GP5 MAb 6D10 and developed using the enhanced chemiluminescence (ECL) method. Binding of EAV VBS to EqCXCL16 protein is shown in (a [arrow]). The same membrane (a) was stripped and re-probed with Gp anti-EqCXCL16 as shown in panel c. As indicated by the arrow in panel c, EqCXCL16S and EqCXCL16R were detected at the same position on the membrane where EAV GP5 was detected in panel a. B). Amino acid substitutions between the “S” and “R” EqCXCL16 isoforms and attachment of EAV to host cells. Equal numbers (2 x 10^6^) of a) naïve HEK-293T cells and stable b) HEK-EqCXCL16S and c) HEK-EqCXCL16R cells were washed, resuspended in cold PBS (pH 7.4) with 2% FBS (PBS-F) and then incubated with biotinylated EAV VBS on ice for 2 h in the dark. After adsorption, excess EAV was removed by washing in cold PBS-F, and cells were then stained with Streptavidin-FITC and DAPI solution. Significantly higher number of HEK-EqCXCL16S cells were observed to bind biotinylated EAV (panel a) compared to the HEK-EqCXCL16R (panel b) or naïve control HEK-293T cells (panel c). Statistically different results are represented with different letters, a and b. Panel d shows the graphical representation of the images from panels a, b, and c. All the images depicted were representative of three independent experiments. Data were analyzed by ANOVA; P<0.001 was considered as significant.

By employing a combination of the virus overlay protein binding assay (VOPBA) and Far-Western blot (Far-WB) techniques, we previously demonstrated that EAV VBS could directly bind with EqCXCL16S [23]. In view of the results outlined above, the experiment was repeated with the stable cell lines expressing both isoforms of EqCXCL16 (HEK-EqCXCL16S and HEK-EqCXCL16R cell lines). Equal amounts of total lysate from HEK-EqCXCL16S, HEK-EqCXCL16R, and mock transfected HEK-293T cells were subjected to polyacrylamide gel electrophoresis and transferred onto a polyvinylidene difluoride (PVDF) membrane prior to sequential denaturation and renaturation by treatment with guanidine-HCl (Gn-HCl) AC buffer. Refolded membrane-bound prey proteins were incubated with purified EAV VBS (bait protein) which was detected using a mouse monoclonal antibody directed against the GP5 envelope protein of EAV (mouse α-GP5 Ab). Two strong signals were detected with apparent molecular weights (MW) of approximately 52 and 30 kDa in HEK-EqCXCL16S, but not HEK-EqCXCL16R or mock transfected HEK-293T (naïve HEK) cell lysates (Fig 3A, panel a). No bands were visible in a control experiment where the EAV VBS binding step was omitted (Fig 3A, panel b), demonstrating the absence of non-specific interactions between mouse α-GP5 Ab and cellular lysate proteins. The monomeric form of CXCL16S has an apparent molecular weight of 30 kDa, and this was confirmed by stripping the membrane depicted in panel a of Fig 3 and re-probing with polyclonal Gp α-EqCXCL16 antisera (pAb). In this experiment, co-migrating bands with an apparent MW of 30 kDa were visible in both HEK-EqCXCL16S and HEK-CXCL16R lysates (Fig 3, panel c). The detection of a strong signal at 52 kDa is unlikely to be the result of interactions between EAV VBS and another cellular protein because this was only visible in HEK-EqCXCL16S lysates. Therefore, while initial polyacrylamide gel separation of the cell lysates was conducted under denaturating conditions, it is conceivable that EqCXCL16S may associate strongly with other proteins. Some support for this hypothesis is provided by the fact Gp α-EqCXCL16 antisera reacts with material with apparent MWs between 52 and 30 kDa in HEK-EqCXCL16S and HEK-EqCXCL16R, but not in mock transfected HEK-293T cell lysates (Fig 3, panel c).

To further confirm these observations, biotinylated EAV VBS was incubated with HEK-EqCXCL16S, HEK-EqCXCL16R, and naïve control HEK-293T cells at 4°C to prevent internalization. Subsequently, the bound virus particles were visualized using streptavidin FITC. Fluorescence microscopy revealed that significantly higher number of HEK-EqCXCL16S cells bound to biotinylated EAV VBS compared to the HEK-EqCXCL16R or control HEK-293T cells (Fig 3B). Taken together, these results (Fig 3A and Fig 3B) confirm that EAV VBS is capable of binding to EqCXCL16S, but that this association is almost completely abrogated by the four amino acid substitutions encoded by exon 1 of *EqCXCL16R*.

### Scavenger receptor activity of EqCXCL16S and EqCXCL16R proteins

**Fig 4:**
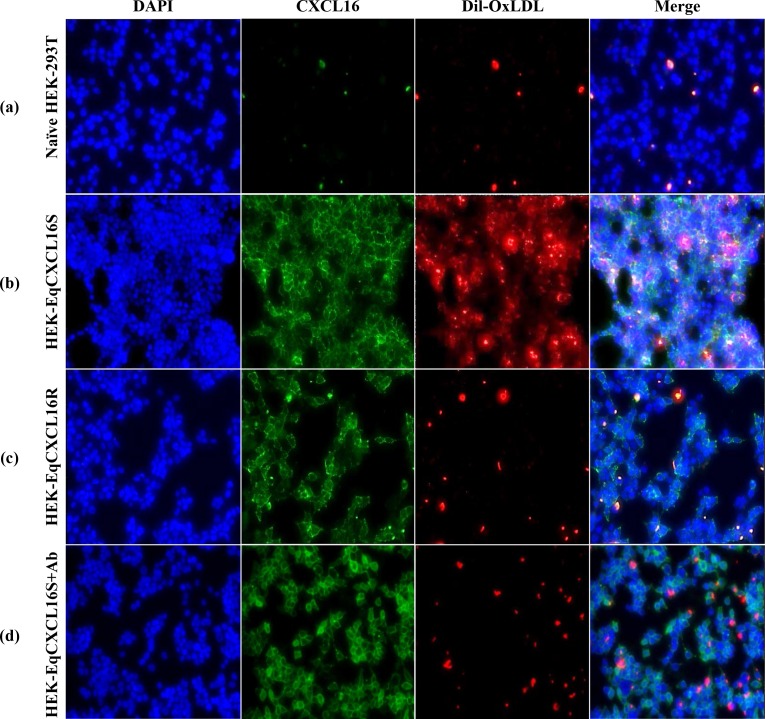
Differences between the membrane-bound forms of each EqCXCL16 isoform in OxLDL scavenger receptor activity. Approximately 5 x 10^5^ naïve HEK-293T and stable HEK-EqCXCL16S and HEK-EqCXCL16R cells/well were plated on a 24-well plate, permitted to adhere for 24 h prior to washing and incubation with Dil-OxLDL for 3 h at 37°C. Following this, cells were washed again with PBS pre-warmed to 37°C, fixed with 4% PFA, and examined with an inverted fluorescence microscope. A marked increase in Dil-OxLDL binding and internalization was shown in HEK-EqCXCL16S (b) as compared to naïve HEK-293T (a), or HEK-EqCXCL16R (c), or HEK-EqCXCL16S cells treated with Gp α-EqCXCL16 pAb (d).

The CXCL16 protein was originally identified as a scavenger receptor for oxidized low-density lipoprotein (OxLDL) in humans [25, 26]. Therefore, it was decided to determine if scavenger receptor activity differed between EqCXCL16 isoforms. The HEK-EqCXCL16S, HEK-EqCXCL16R, and naïve HEK-293T control cells were incubated with Dil labelled OxLDL (Dil-OxLDL) and analyzed by fluorescence microscopy. Interestingly, the highest levels of Dil-OxLDL binding and potential internalization were observed in the HEK-EqCXCL16S while amounts bound to HEK-EqCXCL16R were very low, similar to those observed in mock transfected naïve HEK-293T cells (Fig 4, panel b vs panels a and c). Furthermore, Dil-OxLDL binding to HEK-EqCXCL16S cells was significantly reduced by prior treatment with a polyclonal antibody against EqCXCL16 (Gp α-EqCXCL16 pAb; Fig 4, panel d), suggesting a high degree of specificity in reactivity between Dil-OxLDL and the “S” form of the EqCXCL16 chemokine. These data strongly suggest that the EqCXCL16S isoform retains the function of an efficient scavenger receptor for Dil-OxLDL, whereas this functional property appears to be almost completely absent in the EqCXCL16R isoform. This confirms that four amino acid substitutions in the ectodomain of the EqCXCL16S isoform play a critical role in scavenger properties which may also favor the infection of cells with EAV as compared to the EqCXCL16R isoform.

### Both variants of EqCXCL16 bind to the equine CXC-chemokine receptor 6 (EqCXCR6) protein *in vitro*

**Fig 5:**
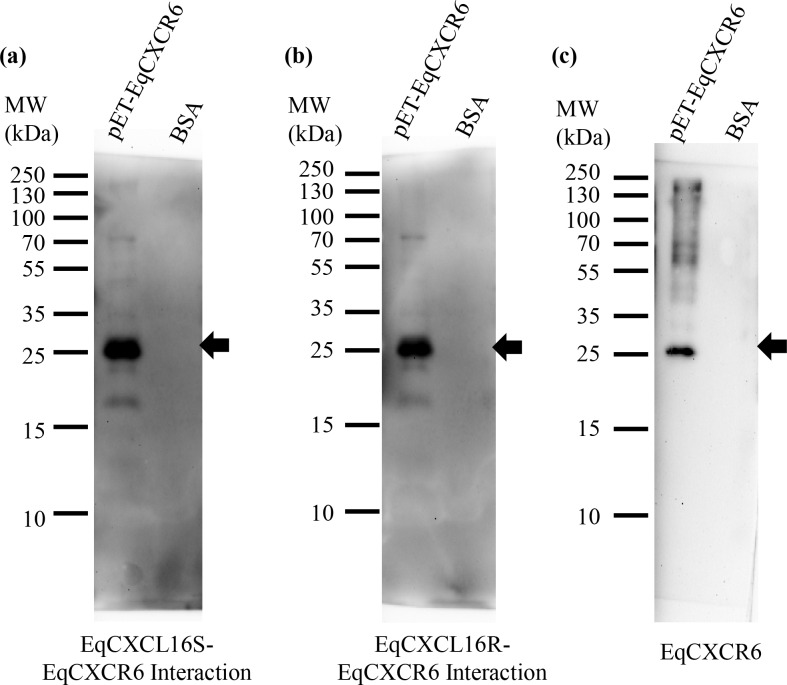
Effect of amino acid substitutions between “S” and “R” isoforms of EqCXCL16 on binding to the EqCXCR6 receptor protein *in vitro*. Interactions between purified recombinant EqCXCL16S/R-EqCXCR6 were examined using Far-WB. Equal amounts (20 μg) of His-tagged EqCXCR6 protein or BSA as a control were separated in different lanes on 10% SDS-PAGE and transferred onto a PVDF membrane. Proteins were then sequentially denatured and renatured by using different concentrations of Gn-HCl. After blocking, the membranes were incubated with soluble EqCXCL16S (panel a) or EqCXCL16R (panel b) protein (5 μg/ml) followed by Rb α-EqCXCL16 Ab. After washing, membranes were developed using the ECL method. Binding of EqCXCL16S and EqCXCL16R to EqCXCR6 is indicated by arrows (panels a and b). These interactions occurred at the same location as that occupied by EqCXCR6; this was confirmed by stripping the membrane shown in panel b and re-probing it with anti-His antibody as shown in panel c.

Although the experiments described above were not quantitative, they did reveal obvious differences between the two EqCXCL16 isoforms in terms of OxLDL scavenger receptor activity. Therefore, Far-WB analysis was employed to determine if similar differences existed between EqCXCL16S and EqCXCL16R in binding to the CXCR6 receptor molecule. Purified recombinant HA-tagged EqCXCR6 protein was separated in SDS-PAGE and incubated with purified recombinant EqCXCL16S or EqCXCL16R protein and binding detected using EqCXCL16 specific antibody produced in rabbits (Rb α-EqCXCL16 Ab). Very similar signal levels were observed with both EqCXCL16 isoforms (Fig 5, panels a and b) at a position approximating a MW of 25 kDa which is the predicted size for cloned EqCXCR6. This was confirmed by stripping the membrane depicted in Fig 5 (panel a) before re-probing with anti-His antibody, thus demonstrating that the location of the His-tagged EqCXCR6 protein was identical to that observed for the interaction between EqCXCL16 and EqCXCR6 (Fig 5, panel c). There was no non-specific reactivity between the EqCXCL16 isoforms and the anti-His antibody with BSA. These data demonstrated there are no major qualitative differences between EqCXCL16S and EqCXCL16R in binding to the EqCXCR6 receptor protein.

### Chemoattractant properties *in vitro* for CD3^+^ T lymphocytes do not differ between the two EqCXCL16 isoforms

**Fig 6:**
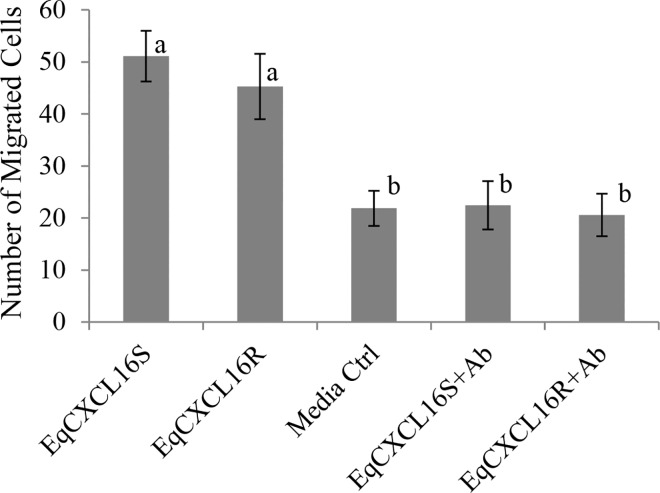
Effect of amino acid substitutions between “S” and “R” isoforms of EqCXCL16 on chemoattractant properties of EqCXCL16S and EqCXCL16R. Purified recombinant soluble forms of EqCXCL16S and EqCXCL16R proteins (2 μg/ml) in RPMI medium containing 0.5% BSA were added to the lower compartment of a Boyden chemotaxis chamber separated from the upper by a polycarbonate filter having a 3 μm pore size; CD3^+^ T lymphocytes (5 x10^5^) labelled with Calcein-AM were added to the upper chamber and incubated for 6 h. The cells that passed through the filter were counted with a fluorescent microscope and represented in a bar diagram. Controls consisted of RPMI with 0.5% BSA and EqCXCL16 (S and R) pre-treated by incubation with Gp α-EqCXCL16 pAb. No statistically significant differences were found in chemoattractant potential between recombinant EqCXCL16S and EqCXCL16R proteins whereas CD3^+^ T lymphocyte migration in response to pre-treatment of these molecules with Gp α-EqCXCL16 pAb was similar to that observed in the medium only control. Experiments were repeated independently three times. The bar diagram represents mean ± SD, P<0.00105 values were considered as significant by ANOVA. Both a and b are significantly different.

The fact that human CXCL16 can act as a chemoattractant and recruit lymphoid cell types to sites of inflammation within the body suggests that this property may also be present in EqCXCL16 [25, 27]. Therefore, experiments to determine if EqCXCL16 possessed chemoattractant potential and if this differed between the two isoforms were conducted using purified recombinant soluble forms of EqCXCL16S and EqCXCL16R proteins produced in *E*.*coli*. Equine CD3^+^ T lymphocytes derived from peripheral blood mononuclear cells (PBMCs) were enriched using anti-CD3 conjugated magnetic beads to > 95% purity based on the proportion of CD4^+^ and CD8^+^ T lymphocytes (S1A Fig top row) present in the population with the remainder comprised mainly of CD21^+^ B cells and CD14^+^ monocytes (S1A Fig bottom row). Approximately 6% of this enriched CD3^+^ T lymphocyte population had detectable cell-surface expression of EqCXCR6 as determined by staining with rabbit anti-EqCXCR6 antibody (PA7511) followed by analysis using flow cytometry (S1B Fig). Incubation of enriched equine CD3^+^ T lymphocyte preparations at 37°C with the recombinant chemokines separated using a Boyden chamber with a 3 μm pore size provided clearest evidence that EqCXCL16 possessed chemoattractant potential compared to the medium only control (P<0.001) (Fig 6). Furthermore, there were no statistically significant differences between the S and R isoforms in numbers of EqCXCR6 expressing CD3^+^ T lymphocytes entering the chamber (P = 0.210) (Fig 6). Evidence of the chemoattractant properties can be attributed directly to EqCXCL16 and not to potential contaminants in the recombinant protein preparations was substantiated by the fact CD3^+^ T lymphocyte migration into the chamber was not statistically different from the medium only control where the equine chemokine isoforms were pre-incubated with Gp α-EqCXCL16 pAb (Fig 6).

### EqCXCL16 as a cellular adhesion molecule

**Fig 7:**
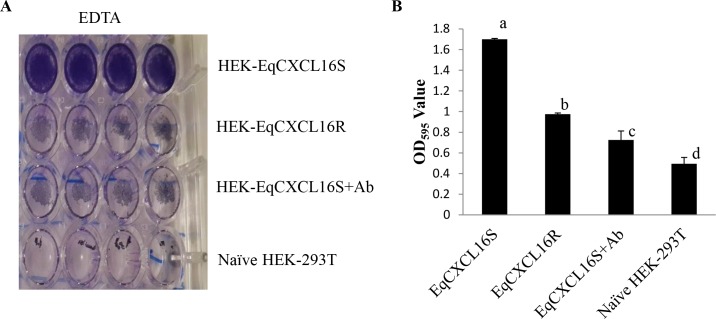
Cellular adhesion properties of EqCXCL16S and EqCXCL16R. Naïve HEK-293T, stable HEK-EqCXCL16S and HEK-EqCXCL16R cells (5 x 10^5^ cells/well) with or without pre-treatment with Gp α-EqCXCL16 pAb were permitted to adhere for 24 h before incubation with 0.5M EDTA for 10 min at 37°C, fixation in 4% PFA, and staining with 0.1% crystal violet. The dye was then extracted with 10% acetic acid prior to determining OD_595nm_ values. A) Representative image of a 24-well plate stained with crystal violet following EDTA treatment showing the differences in adhesion of the different HEK-293T-derived cells. B) Stable HEK-EqCXCL16S cells possess significantly higher adhesion properties that can be eliminated by pre-treatment with Gp α-EqCXCL16 pAb compared to naïve HEK-293T or HEK-EqCXCL16R cells. Mean OD_595nm_ values ± SD of the cell lysate are presented in the bar diagram. P<0.001 was considered as significant; a, b, c, and d are significantly different.

Membrane-associated forms of human CXCL16 have been reported to function as cellular adhesion molecules [25]. Preliminary observations suggested differences between HEK cells expressing the two EqCXCL16 isoforms and adhesion to cell culture plates. Therefore, HEK-EqCXCL16S, HEK-EqCXCL16R, and naïve control HEK-293T cells were tested using a previously described protocol [53] to evaluate resistance to detachment from the cell culture plate following incubation for 10 min at 37°C with 0.5M EDTA. Surprisingly, HEK cells expressing EqCXCL16S were found to be significantly (P<0.0001) more resistant to treatment with EDTA than those expressing EqCXCL16R or naïve control HEK-293T cells (Fig 7A and Fig 7B). Furthermore, the superior adhesion properties of HEK-EqCXCL16S were abrogated by pre-treatment prior to plating with Gp anti-EqCXCL16 polyclonal antibody providing strong evidence of resistance to EDTA-induced detachment was mediated only by the “S” isoform of the chemokine (Fig 7A and Fig 7B).

## Discussion

Previously, we have shown that horses can be categorized into two distinct phenotypic groups based on the susceptibility of their CD3^+^ T lymphocytes to *in vitro* infection with EAV [19, 20]. It was also discovered that stallions with EAV-susceptible CD3^+^ T lymphocytes were at higher risk of becoming long-term carriers following exposure to this virus than those with a resistant CD3^+^ T-cell phenotype [21]. Furthermore, a genome wide association study (GWAS) based on single nucleotide polymorphism (SNP) detection using the Equine SNP50 BeadChip suggested an association between these phenotypic traits and a dominant genetic marker(s) located on equine chromosome 11 (ECA11) between nucleotide positions 49,572,804 and 49,643,932 [20]. In this study, we present genetic and functional data confirming that susceptibility or resistance of CD3^+^ T lymphocytes to *in vitro* EAV infection is indeed under genetic control and can be explained based on mutations found in the chromosome region predicted by GWAS.

Whole genome sequencing of one resistant and two susceptible horses revealed 12 non-synonymous nucleotide substitutions distributed among eight previously annotated genes in the targeted region on ECA11. *CXCL16* was implicated as the putative cause of the phenotype based on tests of the SNPs among 10 horses (8 susceptible and 2 resistant) that excluded the seven other genes. Furthermore, genetic association studies based on typing 240 horses of different breeds demonstrated three alleles of *CXCL16* encoding for two proteins, EqCXCL16R and EqCXCL16S. The gene for EqCXCL16S was dominant and correlated completely with the EAV-susceptible CD3^+^ T lymphocyte phenotype. Specifically, EAV susceptibility of CD3^+^ T lymphocyte population members was conferred by two distinct alleles (*EqCXCL16Sa* and *EqCXCL16Sb*), although the resultant proteins were predicted to possess identical amino acid sequences. These susceptibility alleles exhibited a dominant mode of inheritance, while that associated with CD3^+^ T lymphocyte EAV resistance (*EqCXCL16R*) was recessive, in that the phenotype was only found in equids homozygous for *EqCXCL16R*. At least three of the SNPs located within *EqCXCL16Sa*, *EqCXCL16Sb*, and *EqCXCL16R* are predicted to induce non-conservative or even radical amino acid substitutions within exon 1 of the resultant protein.

If analogous to the human orthologue of *CXCL16*, exon 1 should encode the N-terminal ectodomain of the chemokine and, therefore, play a major role in its biological properties. Although there is to our knowledge no published information concerning the functional properties of EqCXCL16, the human equivalent of this protein (CXCL16) is expressed as a type-I membrane protein and is comprised of several distinct domains (chemokine, extracellular [mucin stalk], hydrophobic transmembrane, and intracellular cytoplasmic) [28]. The protein can also exist in an unbound soluble form resulting either from cleavage with cellular metalloproteinases such as ADAM10 [28] or alternative mRNAsplicing [29]. Membrane-associated forms of human CXCL16 (HuCXCL16) are expressed on dendritic cells, CD14^+^ monocytes/macrophages, CD21^+^ B lymphocytes, endothelial cells, smooth muscle cells, and keratinocytes [30–37]. Moreover, expression on these cell types is upregulated by inflammatory mediators and bacterial lipopolysaccharides [27, 29, 36, 38–40]. Both soluble and membrane-bound forms of HuCXCL16 specifically interact with its receptor CXCR6 (also known as STRL33/BONZO/TYMSTR) expressed on the surface of CD4^+^ and CD8^+^ T lymphocytes, NKT cells, and NK cells [27, 41–43]. Binding to the CXCR6 receptor is facilitated by the mucin-stalk located within the extracellular domain of the molecule [44, 45]. Soluble HuCXCL16 has strong chemotactic potential in that it effectively recruits CXCR6^+^ T lymphocytes to sites of inflammation. The extracellular domain of HuCXCL16 also recognizes oxidized low-density lipoprotein (OxLDL) along with phosphatidylserine; therefore, the protein is multifunctional, acting as a scavenger receptor in addition to possessing chemokine activity [34]. Furthermore, aberrant expression of HuCXCL16 is implicated in the pathogenesis of certain viral infections, arthritis, atherosclerosis, and the metastasis of some cancers [46–50]. Preliminary *in silico* studies suggested that EqCXCL16 possesses a domain structure very similar to its human counterpart including the presence of six cysteine residues within the chemokine domain. However, a Tyr-X-Pro-Val motif in the C-terminal intracellular domain believed to act as a potential substrate for tyrosine kinase phosphorylation in human and mouse variants of the protein is replaced by Tyr-X-Pro-Val in the horse.

Experiments described here demonstrate EqCXCL16 binds to the equine orthologue of *CXCR6* (*EqCXCR6*). In addition, the soluble form of EqCXCL16 has chemotactic activity for CXCR6 expressing T lymphocytes. Although there are no detectable qualitative changes in chemotactic property between the two equine isoforms described here, the predicted amino acid substitutions between EqCXCL16R and EqCXCL16S consisting of Tyr to Phe at position (p) 40, Asp to His at p49, Phe to Ile at p50, and Glu to Lys at p52 (p.Tyr40Phe, p.Asp49His, p.Phe50Ile, and p.Glu52Lys) respectively, had considerable effects on the ability to bind OxLDL. Moreover, there were dramatic differences between HEK-293T cells expressing each of these proteins to adhere to plastic culture vessels in the presence of EDTA. Collectively, these results indicate that EqCXCL16S, in common with its human counterpart, has both chemotactic and scavenger receptor activity, whereas the latter property is likely to be substantially reduced in the EqCXCL16R isoform [51]. However, in terms of EAV, the most significant finding is that while the membrane-bound variant of EqCXCL16S can bind this virus and function as a cellular receptor [28], these properties are completely abrogated in the EqCXCL16R isoform. This suggests amino acid residues located at positions 40 to 52 within the chemokine domain of EqCXCL16 have a direct role in viral attachment via interactions with EAV membrane surface glycoproteins. These studies confirm and extend our previous findings [28] that although some viruses such as HIV and severe acute respiratory syndrome (SARS) virus may specifically interact with CXCL16 to influence the course of viral pathogenesis [32, 33], EAV is at present unique in its ability to utilize this chemokine as a receptor protein. However, the choice of a scavenger receptor as a portal for viral entry does have a precedent among the arteriviruses in that porcine reproductive and respiratory syndrome virus (PRRSV) uses CD163 [48].

**Fig 8:**
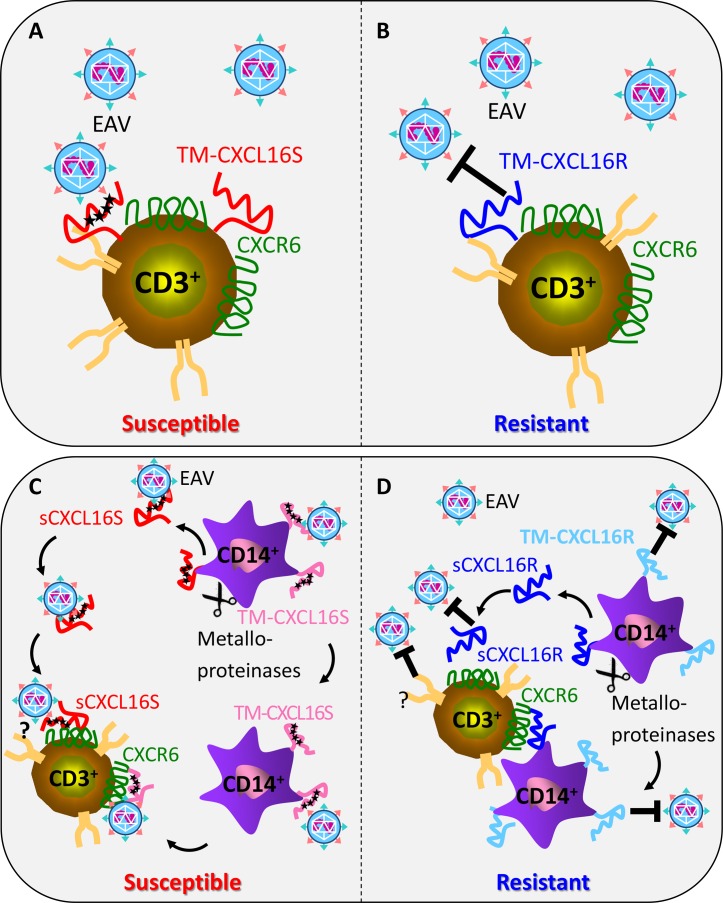
Two models showing the possible mechanisms of EAV infection of equine CD3^+^ T lymphocytes. Model 1: A subpopulation of equine CD3^+^ T lymphocytes expresses transmembrane (TM) form of EqCXCL16S (i.e. horses with the CD3^+^ T lymphocyte subpopulation susceptible to *in vitro* EAV infection; A) or TM-EqCXCL16R (i.e. horses that lack the CD3^+^ T lymphocyte subpopulation susceptible to *in vitro* EAV infection; B) on their cell surface and EAV could directly infect these CD3^+^ T lymphocytes expressing TM-CXCL16S but not those expressing TM-CXCL16R. Model 2: CD3^+^ lymphocytes do not express either isoform of TM-CXCL16 but EAV infects these cells via ligand-receptor (CXCL16-CXCR6) complexes occurring on the CD3^+^ T lymphocytes surface (indirect infection). In this model the CD14^+^ monocytes and macrophages from susceptible and resistant horses express TM-CXCL16S (C) or TM-CXCL16R (D). EAV binds with TM-CXCL16S but not with TM-CXCL16R and is then cleaved by metalloproteinases and the soluble CXCL16S-EAV complex (sCXCL16S-EAV) interacts with CXCR6 present on the CD3^+^ T lymphocytes and thus, infects the CD3^+^ T lymphocytes from susceptible horses (C). It is also possible that TM-CXCL16S after interaction with EAV is not cleaved but comes in contact with CXCR6 present on the CD3^+^ T lymphocytes cells and thus infects the CD3^+^ T lymphocytes. TM-CXCL16R will not bind with EAV and hence CD3^+^ T lymphocytes of resistant horses will not become infected (D).

It has been reported that CXCL16 in humans is expressed by a subpopulation(s) of T lymphocytes [49,50]. Consequently, the simplest explanation for the two phenotypically distinct horse groups identified in our earlier studies [16] is that EAV “susceptible” animals (*EqCXCL16Sa*,*b/EqCXCL16Sa*,*b* or *EqCXCL16Sa*,*b/EqCXCL16R*) possess a subpopulation(s) of T lymphocytes with cell-surface expression of EqCXCL16S and are thus permissive for viral infection, while the equivalent CD3^+^ T lymphocyte population in those that are “resistant” express only EqCXCL16R (*EqCXCL16R/EqCXCL16R*). Further studies are needed to investigate whether EqCXCL16 is constitutively expressed on CD3^+^ T lymphocytes or if expression can be increased by mitogen, TNFα, and IFN**γ** stimulation and/or by EAV infection of equine PBMCs. Another caveat to this hypothesis is that EAV exhibits a broad host-cell tropism and can infect several common laboratory cell lines from different species that certainly do not express EqCXCL16. Previous studies from our laboratory and others have shown that EAV can utilize a number of different non-related, host-specified molecules as cellular entry receptors or accessory molecules [52]. Consequently, demonstrating that susceptibility of a CD3^+^ T lymphocyte subpopulation(s) to EAV infection results from the presence of EqCXCL16S acting directly as an entry receptor will necessitate not only showing the presence of this protein, but also that alternative receptors are either not expressed in these cells or, for some reason, not utilized. Furthermore, the exact mechanism(s) of CXCL16 in infection of equine CD3^+^ T lymphocytes is yet to be determined. One possibility is that CXCL16S (or CXCL16R) is constitutively expressed or can be induced to express on subpopulation of CD3^+^ T lymphocytes, but only a CD3^+^ T lymphocyte subpopulation expressing CXCL16S becomes infected with EAV (Fig 8A and 8B; Model 1). Alternatively, viral entry is mediated by the interaction between soluble isoform of CXCL16S and its cellular receptor CXCR6 expressed on CD3^+^ T lymphocytes (Fig 8C and 8D; Model 2). However, these two models are not mutually exclusive and, as such, both need to be tested to unequivocally confirm the mechanism of CD3^+^ T lymphocyte infection dynamics.

Although differences between horses in EAV CD3^+^ T lymphocyte subpopulation susceptibility is an interesting observation and may correlate with the severity of acute clinical signs [53], it is the association between the *EqCXCL16* genotype and long-term carrier status in stallions that is of major importance because these animals are key to the survival of this virus in equid populations [10]. In contrast to the CD3^+^ T lymphocyte EAV-susceptible/resistant phenotype, the correlation between carrier status in stallions and *EqCXCL16* allelic content was strong (P<0.000001 Fisher's Exact Test without regard to breed) but not absolute. This P-statistic has limited value in that the stallions in Table 6 came from diverse breeds (Table 5), however the association of the genotype with the phenotype without regard to breed was remarkable. In an analysis of 77 EAV-infected stallions, only 14% of the shedders and 86% of non-shedders were homozygous for the resistance genotype. Conversely, 74% of the shedders and 26% of non-shedders had the allele for the EqCXCL16S protein. Although results presented here are consistent with existence of the long-term carrier status in the majority of stallions being dependent on the membrane-bound form of EqCXCL16S acting as a cellular receptor for EAV, the fact that the *EqCXCL16* allelic association is not complete suggests additional genetic, immunological, and viral factors or even environmental factors also play a role in this determination.

Furthermore, the EAV carrier state in stallions has a number of features that distinguish it from many other persistent viral infections of the male reproductive tract. These add additional layers of complexity and must be considered in any proposed mechanism. For example, recent virus isolation and immunohistochemistry studies have confirmed that EAV persists primarily in the ampulla along with other accessory sex glands rather than in immunologically privileged sites such as the Sertoli cells within the testis (Carossino et al. manuscript submitted) [10]. This is despite the fact that carrier stallions possess active immune responses against EAV, and the virus is not detectable in any organ or tissue except the reproductive tract in carrier animals. Indeed inflammatory infiltrates in close proximity to viral antigen-expressing cells are frequently observed in the stallion reproductive tract indicating that EAV persistence is a continuous, dynamic process that occurs in the presence of active local immune responses. Clearly these responses are not completely effective in clearance of the virus for reasons that are unknown at present. Potential explanations range from some form of localized immunosuppression based on the fact that androgens such as testosterone can down-regulate immune responses, to more specific activity such as that mediated by T regulatory lymphocytes or even to differences in functional properties between EqCXCL16S and EqCXCL16R. These are not mutually exclusive, and so the failure to eliminate EAV from the stallion’s reproductive tract could result from a combination of mechanistic factors. In addition, the virus probably contributes to its own survival via antigenic drift as evidenced by the continual emergence of novel variants during the course of persistent infections [16, 54, 55]. However, it is also possible that variation exists between individual stallions in the efficacy of EAV-specific immune responses within the reproductive tract that can operate independently of the *EqCXCL16* genotype. If so, this could explain why 25% of stallions that possess at least one *EqCXCL16Sa/b* allele cease shedding shortly after infection with EAV, while approximately one in six that are homozygous for *EqCXCL16R* become long-term (more than one year) carriers. Based on the *EqCXCL16* gene polymorphisms and its association with long-term carrier status, it would be possible to develop an allelic discrimination real-time PCR assay to distinguish horses that are prone to become long-term versus short-term shedders.

It is interesting that while polymorphisms of *CXCL16* have been reported in, for example, the human *CXCL16* gene [44, 51, 56–58], these are located in regions other than exon 1. Furthermore, these polymorphisms have not been shown to completely abrogate the chemokine, scavenger receptor properties [43]. The horse may therefore be highly unusual in possessing allelic variants of this gene with SNPs situated in exon 1 that in the case of *EqCXCL16R* appear to disrupt the ability of the resultant protein to act as a scavenger receptor for OxLDL.

In conclusion, these genomic studies unequivocally demonstrate that horse genomic sequences encoding the EqCXCL16 chemokine are associated with *in vitro* susceptibility of equine CD3^+^ T lymphocytes to EAV infection, as well as the establishment of long-term carrier state in stallions. Although the molecular mechanisms associated with these phenotypic traits have not been fully elucidated, there is compelling evidence the plasma membrane-associated variant of EqCXCL16S can function as a cellular entry receptor for EAV, whereas this property is absent in the EqCXCL16R isoform of the protein. It is interesting that the sequence associated with abrogation of the virus binding site costs the horse the scavenging capability of CXCL16. If we assume the *EqCXCL16S*a/b form is ancestral, the virus will have made use of a functional part of EqCXCL16, potentially to deter adaptation; selective pressure may have been sufficient for evolution of a variant that does not allow virus binding at the cost of losing the scavenging capability for OxLDL. The origins and selection pressures for these variants of *EqCXCL16* warrant further study.

## Materials and Methods

### Ethics statement

This study was performed in strict accordance with the recommendations in the Guide for the Care and Use of Laboratory Animals of the National Institutes of Health. The animal protocol involving horses was approved by the University of Kentucky Institutional Animal Care and Use Committee (IACUC; protocol number 2013–1098). The animal protocol involving rabbits and guinea pigs was approved by the Thermo Scientific, Rockford, IL IACUC (NIH OLAW assurance number: A3669-01, USDA research license registration number: 23-R-0089, and PHS assurance number: A3669-01). This study was performed according to these IACUC-approved protocols.

### Cells

Equine pulmonary artery endothelial cells (EECs) were maintained in Dulbecco’s modified Eagle’s medium (DMEM; Mediatech, Herndon, VA, USA) with sodium pyruvate, 10% fetal bovine serum (Hyclone Laboratories, Inc., Logan, UT, USA), 100 U/ml of penicillin/100 μg/ml of streptomycin (Gibco, Carlsbad, CA, USA), and 200 mM L-glutamine [59–61]. The high passaged rabbit kidney cells (HP-RK-13 [KY] P399-409, originally derived from ATCC CCL-37; American Type Culture Collection, Manassas, VA, USA) were propagated in Eagle’s minimal essential medium with 10% ferritin-supplemented bovine calf serum (Hyclone Laboratories, Inc.) and 100 U/ml of penicillin/100 μg/ml of streptomycin (Gibco). Human embryonic kidney (HEK-293T) cells (ATCC CRL-3216) were propagated in DMEM with 10% ferritin-supplemented bovine calf serum (Hyclone Laboratories, Inc.),100 U/ml of penicillin, and 100μg/ml streptomycin (Gibco). HEK-293T cells stably expressing EqCXCL16S and EqCXCL16R were maintained in DMEM with 10% ferritin-supplemented bovine calf serum (Hyclone Laboratories, Inc.) and puromycin (Clontech Laboratories Inc., Mountain View, CA, USA) at 3 μg/ml of medium.

### Isolation of peripheral blood mononuclear cells (PBMCs) and enrichment of equine CD3^+^ T lymphocytes

Isolation of PBMCs from peripheral blood of horses (n = 9) was performed as described previously [19, 45] with some modifications. Briefly, blood (20 ml) was collected aseptically using Vacutainer tubes containing 0.1 ml of 15% EDTA solution (Covidien, Dublin, Ireland). PBMCs were isolated from the buffy-coat fraction by centrifugation through Ficoll-Paque Plus (Amersham Biosciences, Piscataway, NJ, USA) at 500 × g for 30 min at 25°C. The PBMC layer was collected and washed twice with Hanks balanced salt solution (pH 7.4) (Life Technologies, Grand Island, NY, USA) by centrifugation at 300 × g for 10 min to eliminate the platelets. The cells were resuspended in RPMI-1640 medium with 2 mM GlutaMAX and 25 mM HEPES (Life Technologies) without FBS and counted using a Countess Automated Cell Counter (Life Technologies). CD3^+^ T lymphocytes were enriched indirectly using magnetic microbeads according to the manufacturer’s protocol (Miltenyi Biotec, Bergisch Gladbach, Germany). Briefly, PBMCs were blocked with 5% normal mouse serum (Innovative Research, Novi, MI, USA) and then incubated with anti-equine CD3 (Clone UC F6G) conjugated with Alexa Fluor 647 for 30 min. After washing with MACS buffer, the cells were incubated with anti-Alexa Fluor 647 microbeads for 20 min. The cells were then washed with MACS buffer and applied to an LS column, washed three times, and the bound CD3^+^ T lymphocytes were eluted with MACS buffer in the absence of the magnet, washed, and counted using a Countess Automated Cell Counter. The purity of the CD3^+^ T lymphocytes was confirmed by flow cytometric analysis. Staining was performed following the protocol as described previously [20, 45]. These cells were used for studying the chemokine function of EqCXCL16S and EqCXCL16R.

### Viruses

Two strains of EAV, the experimentally derived virulent Bucyrus strain (VBS) (ATCC VR-796) [4, 62] and the recombinant EAV VBS virus expressing mCherry (EAV sVBSmCherry) [24] were used. Both viruses were propagated in EECs to generate high titer working stocks as previously described [63, 64]. Briefly, EECs infected with each virus were frozen at -80°C when 90–100% cytopathic effect (CPE) was observed. Cell lysates were clarified by centrifugation (500 × g) at 4°C for 15 min, followed by ultracentrifugation (Beckman Coulter, Miami, FL, USA) at 121,600 × g through a 20% sucrose cushion in NET buffer (150 mM NaCl, 5 mM EDTA, and 50 mM Tris-HCl, pH 7.5) at 4°C for 4 h to pellet the virus. Purified preparations of each strain of EAV were resuspended in phosphate buffered saline (PBS, pH 7.4) and frozen at -80°C. Virus stocks were titrated by standard plaque assay in RK-13 cells and titers were expressed as PFU/ml [65].

### Horses for phenotypic and genotypic testing

A total of 240 horses from four different breeds, Thoroughbred (TB; n = 67), American Saddlebred (ASB; n = 60), Standardbred (STB; n = 60), and Quarter Horses (QH; n = 53) were randomly selected for sequencing. These horses were a random subset of horses randomly selected from farms in Central Kentucky and previously tested for CD3^+^ T lymphocyte phenotyping [15].

### Semen from EAV carrier and non-carrier stallions

A panel of 77 archived semen samples from EAV carrier stallions (n = 37) and non-carrier (n = 40) that had been stored at -80°C was tested. These semen samples were previously submitted to the EVA OIE Reference Laboratory at the Maxwell H. Gluck Equine Research Center, Lexington, KY for testing. To confirm carrier status, isolation of EAV from equine semen samples was attempted in a high passage (P399-409) rabbit kidney-13 (RK-13) (HP-RK-13 [KY]) cell line according to the OIE described protocol [66]. Serum neutralizing antibodies to EAV were demonstrated by microneutralization assay in both carrier and non-carrier stallions to confirm that they were seropositive for EAV [67]. Based on clinical histories, none of the stallions had been vaccinated against EAV, and seroconversion was the result of natural infection. EAV long-term shedders were defined as those horses that had detectable EAV in their semen for more than one year following infection where date of exposure was known. Non-shedders of EAV were those which likely shed EAV in their semen during the acute phase of infection but that had ceased shedding virus at the time of initial testing for presence of the carrier state. These horses came from a wide range of breeds including Warmbloods, Standardbreds, Thoroughbreds, Quarter Horses, Belgian draft, Andalusian, Friesian, Rocky Mountain, Selle Francais, Tennessee Walking Horse, Arabian, Lusitano and American Saddlebred horses (Table 5). Comparisons of shedders to non-shedders for EqCXCL16 genotypes were made using Fisher's Exact Test.

### Phenotypic trait

The susceptible or resistant phenotype of each animal was defined by dual-color flow cytometric analysis of *in vitro* EAV-infected CD3^+^ T lymphocytes as described previously [28]. Horses were classified as susceptible or resistant to *in vitro* EAV infection based on the *in vitro* susceptibility or resistance of their CD3^+^ T lymphocytes.

### DNA extraction

Genomic DNA (gDNA) was obtained from PBMCs or semen of each animal by using the Puregene whole-blood extraction kit (Qiagen, Valencia, CA, USA) in accordance with the manufacturer’s instructions as previously described [20]. DNA quality and concentration were assessed using Nanodrop (Thermo Scientific, Wilmington, DE, USA) at an absorbance ratio of optical density at 260 nm/280 nm (OD260/280).

### Next-generation sequencing of complete genome

Three horses were selected from those SNP genotyped and phenotyped for the CD3^+^ T lymphocyte susceptibility (S) or resistance (R) phenotype based on dual-color flow cytometric analysis in the previous study [19]; specifically, they were TB03 (R), TB10 (S), and ST22 (S). Genomic DNA was submitted to BGI Americas (Davis, CA, USA) for sequencing from each of these three horses. Approximately 20 μg of DNA was submitted for construction of short insert (500 bp) libraries for sequencing using the Illumina HiSeq2000. To achieve 20–35 GB of raw data per lane,100 bp paired end sequencing across 7 lanes was conducted. Approximately 30× coverage was obtained per sample; there were 874,258,138 clean reads of 95.86 Q20 with ST22; 838,009,546 clean reads of 95.61 Q20 with TB03; and 867,086,552 clean reads of 95.96 Q20 with TB10. Reads were mapped to the horse genome reference sequence (Ecab 2.0) [68] using CLC workbench 8.0.1(CLC Bio, Boston, MA, USA). The whole genome sequence data from the three horses have been submitted to the Sequence Read Archive and can be found under BioSample/experiment accession numbers SAMN03838869/SRX1097022, SAMN03838867/ SRX1097495 and SAMN03838868/SRX1097492 for CXCL16 of TB10, TB3, and ST22, horses respectively. The variant discovery and genotyping were done with the Genome Analysis Toolkit UnifiedGenotyper using arguments -nt 4 -gt_mode DISCOVERY—validation_strictness LENIENT.[69]. Genome annotation was from Ensembl version *Equus caballus*. EquCab2.75.

### DNA sequencing of candidate genes

The primer sequences for the eight candidate genes found using the target region (ECA11: 48M-51M) are shown in S2 Table. A PCR was performed in a final volume of 18 μL and the reaction for amplification consisted of: 100 ng of genomic DNA, 0.15 mM of each primer, and 10 μL of AmpliTaq GoldFast PCR Master Mix (Applied Biosystems, Foster City, CA, USA). Amplifications consisted of the following steps: initial denaturation at 95°C for 10 min; 30 cycles of denaturation at 95°C for 30 sec, annealing of primers at 56°C for 30 sec, extension at 72°C for 30 sec; and a final extension at 72°C for 10 min. Amplicons were shipped overnight for sequencing to Eurofins Genomics (Louisville, KY, USA). DNA sequences were analyzed using Chromas Lite (Technelysium Pty Ltd, South Brisbane, Australia). For annotation of bases for equine *CXCL16*, the sequence XM_001504756 was used.

### Species comparisons for CXCL16 protein

The NCBI database was used to obtain and compare reference genome predicted-protein sequences of CXCL16 for white rhinoceros (*Ceratotherium simum;* XM_004433427), horse (*Equus caballus;* XM_001504756), dog (*Canis lupus familiaris;* XM_844211), human (*Homo sapiens*; AY358909), domestic rat (*Rattus norvegicus;* DQ025528), cattle (*Bos taurus*; NM_001046095) and African elephant (*Loxodonta Africana;* XM_003416737).

### Cloning and expression of EqCXCL16S, EqCXCL16R, and EqCXCR6 in *E*. *coli*

Recombinant plasmids for cloning EqCXCL16 and EqCXCR6 were designed by CLC Main Workbench 7 programs using horse genome sequences obtained as part of this study. Synthetic sequences encoding the predicted exposed part of EqCXCL16 (aa 25–199) [45] and the entire EqCXCR6 were produced by IDT (Coralville, IA, USA). These sequences were designed to have flanking *BamHI* and *XhoI* sites. In order to produce recombinant plasmids encoding the full size of S and R versions of CXCL16, synthetic sequences flanked by *PstI* and *XhoI* were also produced. These sequences encoded the N terminus half (aa 1–143) of both versions of EqCXCL16 (S and R). Following digestion with *BamHI* and *XhoI* or *PstI* and *XhoI* (Thermo Scientific, Rockford, IL, USA), fragments of amplicon and synthetic DNA were separated in E-Gel EX 1% agarose (Life Technologies) and extracted from gel using Zymoclean Gel Recovery Kit (Zymo Research, Irvine, CA, USA). Purified fragments encoding aa 17–247 and aa 25–199 of EqCXCL16 and entire EqCXCR6 were ligated into pET15b (Novagen, Temecula, CA, USA) followed by transformation into *E*. *coli* NovaBlue (Novagen). Ligation and transformation were performed using Rapid DNA Ligation and TransformAid kits (Thermo Scientific), respectively. Recombinant plasmids p15-16A (aa 17–247), p15-16B (aa 25–199), and p15-R6 were isolated from ampicillin-resistant clones using ZR Plasmid Miniprep™ Kit (Zymo Research). Plasmids p15-16R and p15-16S encoding R and S versions of full EqCXCL16 were produced by substitution of the smaller *PstI/XhoI* fragment of p15-16A to synthetic sequences encoding an N terminal half of R and S versions of this protein, respectively. In order to express recombinant polypeptides, plasmids were transformed into E.* coli* BL21(DE3) expression vector (Novagen). Several ampicillin-resistant clones were grown overnight in 1 ml of MagicMedia (Life Technologies) supplemented with 50 μg/ml of ampicillin (Sigma-Aldrich). Production of polypeptides was confirmed by SDS-PAGE electrophoresis following mini-scale isolation of recombinant proteins using Talon Metal Affinity Resin (Clontech Laboratories Inc.). For large scale production of recombinant EqCXCL16 (S and R) and EqCXCR6, 500 ml cultures of BL21(DE3) strain of *E*. *coli* with respective plasmids were grown overnight at 37°C in MagicMedia supplemented with 50 μg/ml of ampicillin. Following centrifugation at 6000 × g for 15 min, the cell pellet was resuspended in Buffer A (50 mM sodium phosphate, 6 M guanidine-HCl, and 300 mM NaCl; pH 7.0) and subjected to several short cycles of sonication to reduce viscosity. The lysate was centrifuged at 16,000  × g for 30 min at 4°C to remove debris. The His-tagged recombinant EqCXCL16 and EqCXCR6 proteins were purified from the supernatant by affinity chromatography using Talon Superflow Metal Affinity Resin (Clontech Laboratories Inc.) in combination with an FPLC apparatus (Amersham Pharmacia Biotech Inc.). Columns were equilibrated and washed with Buffer A, while proteins were eluted using Buffer B (45 mM sodium phosphate, 5.4 mM Gn-HCl, 270 mM NaCl, and 150 mM imidazole; pH 7.0). Eluted recombinant protein was dialyzed against PBS using Slide-A-Lyzer Dialysis Cassettes (Thermo Scientific). The purity and integrity of recombinant EqCXCL16S, EqCXCL16R, and EqCXCR6 proteins were evaluated by subjecting them to electrophoresis on a 4–20% gradient gel SDS followed by staining with PageBlue Protein Staining Solution (Thermo Scientific) and WB analysis. Protein concentration was determined with BCA Protein Assay (Thermo Scientific) using BSA as the standard.

### Antibodies

Protein-specific rabbit antipeptide sera (Rb α-EqCXCL16 [rabbit Ab, PA7509]), and guinea pig polyclonal antibody (Gp α-EqCXCL16 pAb) to detect EqCXCL16 proteins (EqCXCL16S and EqCXCL16R) were generated by immunizing rabbits with two synthetic peptides and guinea pigs with recombinant EqCXCL16 expressed in *E*.*coli* as previously described [45]. For this study anti-EqCXCR6 peptide antibody was generated by immunizing two rabbits with the 14 amino acid peptide (amino acid residues 17–30: DSSQEHERFLQFKK). These antibodies were extensively characterized by ELISA, confocal microscopy, and WB analysis. The monoclonal antibody (MAb) to equine CD3 surface molecule, UC F6G, was kindly provided by Dr. Jeff Stott, University of California, Davis. The R-PE conjugated F(ab′)_2_ fragment of goat anti-mouse IgG1 (Southern Biotech, Birmingham, AL, USA) was used as the secondary antibody. Mouse EAV α-GP5 and mouse EAV α-nsp-1 monoclonal Abs (MAb 6D10 and MAb 12A4, respectively) have been described previously [70, 71]. Detection of EAV antigen in infected cells was conducted using Alexa Fluor 488-labeled MAb against nonstructural protein 1 (nsp1; MAb 12A4) [19, 70]. Goat α-rabbit IgG (H+L)-HRP and goat α-mouse IgG (H+L)-HRP were purchased from Cell Signaling Technology, Inc. (Danvers, MA, USA). Goat α-guinea pig IgG (H+L)-HRP, goat α-rabbit IgG (H+L) conjugated to Alexa Fluor 488, and goat α-guinea pig conjugated to Alexa Fluor 488 were purchased from Life Technologies. Streptavidin conjugated to FITC was purchased from Southern Biotech.

### Establishment of stable HEK-293T cells expressing EqCXCL16S and EqCXCL16R (HEK-EqCXCL16S and HEK-EqCXCL16R)

Stable HEK-293T transfectants expressing EqCXCL16S (HEK-EqCXCL16S cells) and EqCXCL16R (HEK-EqCXCL16R cells) were generated as described earlier [45]. Briefly, for expression of the EqCXCL16R protein in eukaryotic cells, a codon-optimized full-length EqCXCL16R sequence (obtained from whole genome sequencing in this study) was commercially synthesized and cloned into the pJ609 plasmid, into which the puromycin resistance gene was incorporated, by DNA2.0 (Menlo Park, CA, USA). This molecular construct was identified as pJ609-EqCXCL16R and used to transform *E*. *coli* DH10B cells (Life Technologies).

For the establishment of stable cells, the HEK-293T cells were seeded in 6-well plates (2 x 10^6^ cells/well) and transfected with 3 μg of codon-optimized pJ609-EqCXCL16R plasmid DNA (DNA2.0) using lipofectamine 3000 (Life Technologies) following the manufacturer’s instructions. At 24 h post transfection, the medium was replaced with fresh medium containing 4 μg/ml of puromycin (Clontech Laboratories Inc.) and cells were incubated at 37°C in a 5% CO_2_ incubator. This process was repeated every other day until only puromycin-resistant colonies remained. These puromycin resistant cells were cloned by limiting dilution in 96-well plates and screened by IFA, after which clones showing the highest level of EqCXCL16S and EqCXCL16R protein expression were frozen in commercial cell-freezing medium (Recovery Cell Culture Freezing Medium; Life Technologies) and stored in liquid nitrogen until needed. At every 5^th^ passage and up to the 50^th^ serial passage, cells were analyzed by IFA using Gp α-EqCXCL16 pAb to confirm the expression of EqCXCL16R. All the experiments in HEK-EqCXCL16S and HEK-EqCXCL16R cells were performed within passage levels 5 to 10.

### Confocal and indirect immunofluorescence microscopy

Naïve HEK-293T and stable HEK-EqCXCL16S and HEK-EqCXCL16R cells in 8-well Thermo Scientific Lab-TeK chamber slides were washed in cold phosphate buffered saline (PBS, pH 7.4) and fixed in 4% paraformaldehyde (PFA; Sigma-Aldrich, St Louis, MO, USA) for 30 min at room temperature (RT). Cells were then stained as described previously [72]. Following fixation, cells were washed 5 times in ice-cold 10 mM glycine (Sigma-Aldrich) in PBS, pH 7.4 (PBS-Glycine) and were then permeabilized with 0.2% saponin (Sigma-Aldrich) in PBS or left untreated with detergent where examination of surface staining was required. All cells were washed again in 10 mM PBS-glycine and blocked with 5% normal goat serum (MP Biomedicals, Santa Ana, CA, USA) for 30 min at RT prior to incubation with specific primary antibodies (1:100 dilution) for 1 h at 37°C in a humidified chamber. After washing in 10mM PBS-glycine, the cells were incubated with anti-mouse or anti-guinea pig IgG(H+L) secondary antibodies conjugated with Alexa Fluor 488 (AF488, 1:200 dilution) for 1 h at 37°C in a humidified chamber maintained in total darkness. After washing, slides were mounted in Vectashield mounting medium containing 4′, 6-diamidino-2-phenylindole (DAPI; Vector Laboratories, Burlingame, CA, USA). The slides were observed either under a Leica TSP SP5 confocal microscope in an environmental chamber at the University of Kentucky imaging core facility or with an inverted fluorescence microscope (ECLIPSE Ti; Nikon, Melville, NY, USA).

### SDS-PAGE and western immunoblotting

Cells were lysed in RIPA lysis buffer (Santa Cruz Biotechnology, Dallas, TX, USA) in Halt protease and phosphatase inhibitor cocktails (Thermo Scientific). The solubilized proteins were mixed with Pierce lane marker reducing 5X sample buffer containing 100 mM dithiothreitol (DTT; Thermo Scientific) and heated for 5 min at 95°C. Samples were resolved in SDS-polyacrylamide gel (5% stacking and 12% resolving; Bio-Rad) at 200 V for 45 min and then transferred onto a PVDF membrane (Bio-Rad, Hercules, CA, USA) at 100 V for 1 h using the trans-blot transfer system (Bio-Rad) [73, 74]. The membrane was blocked with 5% non-fat milk powder (Bio-Rad) in TBS-T (10 mM Tris-HCl [pH 7.6], 150 mM NaCl, and 0.1% Tween 20) for 1 h at RT and incubated with primary antibodies (Abs): rabbit α-EqCXCL16 PA7509 (1:500), guinea pig α-EqCXCL16 (1:1000), mouse monoclonal α-EAV GP5 MAb 6D10 (1:2000), and mouse monoclonal α-EAV nsp-1Mab (12A4). The Abs were diluted in TBS-T with 5% bovine serum albumin (Sigma-Aldrich) overnight at 4°C. The following day, the membranes were washed with TBS-T and then incubated with anti-rabbit, anti-mouse, or anti-guinea pig IgG, as appropriate, and conjugated with horseradish peroxidase (HRP, 1:3000; Cell Signaling Technology, Inc.) for 1 h at RT. The membranes were washed again and antibody binding was visualized with an ECL-detection system using SuperSignal West Pico chemiluminescent substrate (Thermo Scientific).

### Cell adhesion assay

The cell adhesion assay was performed in accordance with a published protocol [75]. Approximately 1 x 10^5^ HEK-EqCXCL16S, HEK-EqCXCL16R, and naïve HEK-293T cells were plated in a 96-well plate. After 24 h incubation, the cells were washed in 37°C PBS. Cells were then incubated with 0.5M EDTA for 10 min at 37°C following which they were washed again with PBS and fixed with 4% PFA for 15 min at RT. Cells were then washed in distilled water and stained with 0.1% crystal violet solution for 20 min at RT, visualized with an inverted light microscope and photographed. After washing with distilled water, the cells were air-dried, then incubated with 10% acetic acid for 20 min with shaking prior to the transfer of 50 μl of the lysate to a new 96-well ELISA plate for determination of OD_595nm_ values using a Synergy H1MD microplate reader (BioTek Instruments Inc., Winooski, VT, USA).

### Chemokine assay

Cell migration in response to EqCXCL16S and EqCXCL16R proteins was determined using a Chemotaxis assay kit (Cell Biolabs, Inc., San Diego, CA, USA). Briefly, FPLC purified soluble recombinant EqCXCL16S and EqCXCL16R proteins were diluted in serum-free RPMI medium with 0.5% BSA (cell culture grade) at a concentration of 2 μg/ml in 500 μl of RPMI and were added to the lower well of a 24-well chemotaxis chamber; the lower and the upper wells were separated by a polyvinylpyrrolidone-free polycarbonate filter insert with the pore size of 3 μm. To the upper wells of the chamber, 100 μl of purified equine CD3^+^ T lymphocytes (5 x 10^5^ cells/ml) labelled with Calcein-AM (Life Technologies) was added. After 6 h of incubation at 37°C in 5% CO_2_, media from the inside of the insert was removed and insert was placed in a clean well with 400 μl of Cell Detachment solution and incubated at 37°C for 30 min. After complete dislodging of the cells from the underside of the insert, 400 μl of the cells containing Cell Detachment solution was mixed with 400 μl of the migratory cells from the original well. Then 50 μl of the mixed cell solution was added onto a glass slide, and cells that passed through the filter were counted under a fluorescent microscope (40× objective) and represented in a bar diagram (averages of six different fields were included).

### Dil-OxLDL uptake assay

Stable HEK-293T cells expressing EqCXCL16S and EqCXCL16R and naïve HEK-293T cells were seeded in 12-well plates. At about 85% confluency, cells were washed with warm (37°C) PBS (pH 7.4) and replenished with warm (37°C) growth medium containing Dil-OxLDL at 10 μg/ml (Kalen Biomedical LLC, Montgomery Village, MD, USA) and incubated at 37°C for 3 h. Cells were washed with ice-cold PBS and examined under an inverted epifluorescence microscope for the uptake of Dil-OxLDL by cells.

### Virus overlay protein binding assay (VOPBA) and Far-Western blot (Far-WB)

Approximately 100 μg of total protein lysate from naïve HEK-293T cells or stable HEK-EqCXCL16 cells were separated in 12% SDS-PAGE and transferred onto a PVDF membrane for Far-WB analysis following a modification of the published protocol [76]. The bound proteins were then denatured and gradually renatured on the membrane by sequential incubation with 6 M, 3 M, 1 M, and 0.1 M Gn-HCl in freshly prepared AC buffer (100 mM NaCl, 20 mM TRIS [pH 7.5], 10% glycerol, 0.5 mM EDTA, 0.1% Tween-20, 2% non-fat dry milk, and 5 mM DTT) for 30 min at RT or with only AC buffer in the absence of Gn-HCl overnight at 4°C. The membrane was blocked with 5% non-fat dry milk in TBS-T (0.1% Tween-20) and overlaid with purified EAV VBS (15 μg/ml) and incubated overnight at 4°C. The next day, the membrane was washed vigorously (3 washes each of 10 min) and incubated with mouse monoclonal Ab (α-GP5; MAb 6D10) directed against EAV GP5 envelope glycoprotein. Monoclonal antibody binding was detected by the ECL-detection system using SuperSignal West Pico chemiluminescent substrate (Thermo Scientific). To study EqCXCL16-EqCXCR6 interaction in a separate Far-Western blot experiment, 20 μg of purified recombinant hemagglutinin (HA)-tagged EqCXCR6 was separated on 12% SDS-PAGE and transferred onto a PVDF membrane which was incubated with recombinant purified EqCXCL16S or EqCXCL16R proteins (5 μg/ml). The membrane was then developed using Rb-anti EqCXCL16 antibody.

### Labeling of EAV with biotin and EAV binding assay

EAV VBS was purified by ultracentrifugation (121,600 × g for 4 h) through a 20% sucrose cushion and protein concentration determined using the BCA protein assay kit. About 2 mg of purified EAV was biotinylated using EZ-Link Sulfo-NHS-Biotin (Thermo Scientific) following the manufacturer’s protocol. Excess unbound biotin was removed by filtering through a Zeba desalt spin column (MWCO 7000; Thermo Scientific) equilibrated in PBS (pH 7.4). The naïve HEK-293T and HEK-EqCXCL16 cells were washed in cold PBS (pH 7.4) and removed from the culture dish using a non-enzymatic cell dissociation solution (Cellstripper; Mediatech Inc.). Cells were resuspended in cold PBS (pH 7.4) containing 2% FBS (PBS-F), centrifuged at 1000 × g for 5 min at 4°C and incubated with biotinylated EAV at an MOI of 100 on ice for 2 h in total darkness. Excess EAV was removed by washing three times in cold PBS-F. Subsequently, the cells were stained with Streptavidin-FITC (1:100) and incubated at 4°C for 30 min in total darkness. Cells were washed in PBS-F at 1000 × g for 5 min at 4°C, transferred onto glass microscope slides using a Shandon CytoSpin III Cytocentrifuge with Shandon single cytofunnel with white filter cards (Thermo Scientific) and incubated with DAPI solution to permit visualization of cell nuclei. Cells were then analyzed using a Nikon inverted fluorescence microscope and the percentage of cells bound to EAV was calculated.

### Statistical analyses of data

Statistical tests for association of *CXCL16* genotypes with phenotypes, including susceptibility and resistance as well as carrier status, were conducted using the Fisher’s Exact Test. Differences among multiple treatment groups were analyzed by statistical analysis software Sigmaplot 12.3 (SystatSoftware Inc., San Jose, CA, USA), by ANOVA with pairwise multiple comparison procedures by the Holm-Sidak method. P-values less than 0.05 were considered to be statistically significant.

## Supporting Information

S1 Fig A) Enrichment of CD3^+^ T lymphocytes from PBMCs. T lymphocytes were enriched from PBMCs using anti-equine CD3 (Clone UC F6G) and micro magnetic beads conjugated to anti-mouse IgG1. The enriched cells were found to be > 95% T lymphocytes based on the proportion of CD4^+^ and CD8^+^ cells (top row) present in the enriched fraction. Furthermore, both CD21^+^ B lymphocytes and CD14^+^ monocytes (bottom row) were found to be a very minor component of the enriched T lymphocyte fraction. B) Expression of CXCR6 on equine CD3^+^ T lymphocytes. Magnetic bead purified CD3^+^ T lymphocytes were stained with α-EqCXCR6 antibody. Percentage of CD3^+^ T lymphocytes cells stained by pre-bleed rabbit sera as a negative control (shown as a). Percentage of CD3^+^ T lymphocytes expressing CXCR6 (shown as b).
(TIF)

S1 Table Ensembl and NCBI gene identification numbers. (DOCX)

S2 Table Primers used in PCR amplification and sequencing of equine genes located in ECA11. (DOCX)
