# Supplementary figures and images for: Allelic Variation in CXCL16 Determines CD3+ T Lymphocyte Susceptibility to Equine Arteritis Virus Infection and Establishment of Long-Term Carrier State in the Stallion
Source: PLoS Genet. 2016 Dec 8;12(12):e1006467. doi: 10.1371/journal.pgen.1006467 (PMC5145142; doi:10.1371/journal.pgen.1006467)

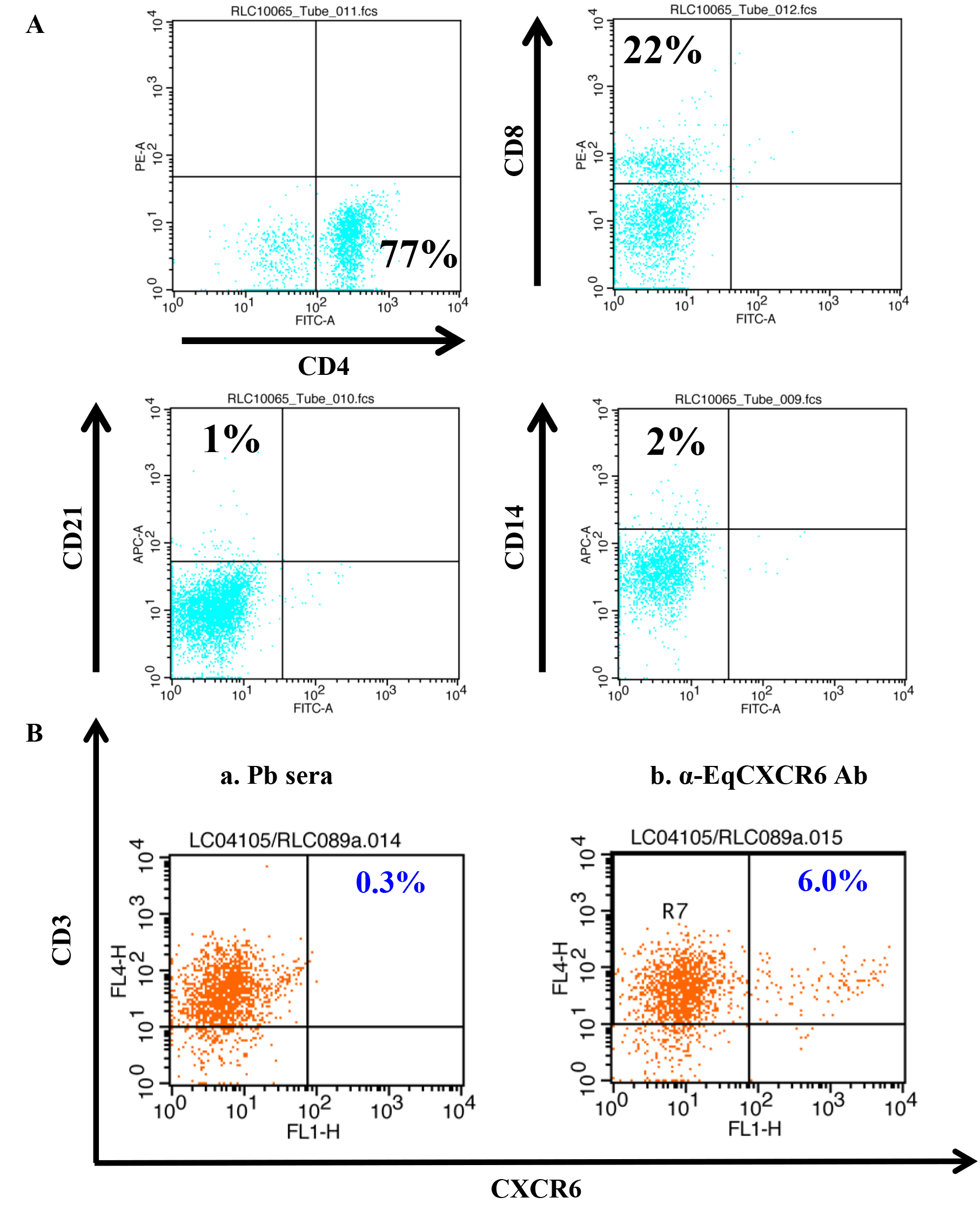

Supplement: S1 Fig — A) Enrichment of CD3+ T lymphocytes from PBMCs. T lymphocytes were enriched from PBMCs using anti-equine CD3 (Clone UC F6G) and micro magnetic beads conjugated to anti-mouse IgG1. The enriched cells were found to be > 95% T lymphocytes based on the proportion of CD4+ and CD8+ cells (top row) present in the enriched fraction. Furthermore, both CD21+ B lymphocytes and CD14+ monocytes (bottom row) were found to be a very minor component of the enriched T lymphocyte fraction. B) Expression of CXCR6 on equine CD3+ T lymphocytes. Magnetic bead purified CD3+ T lymphocytes were stained with α-EqCXCR6 antibody. Percentage of CD3+ T lymphocytes cells stained by pre-bleed rabbit sera as a negative control (shown as a). Percentage of CD3+ T lymphocytes expressing CXCR6 (shown as b). (TIF) [file pgen.1006467.s001.tif]
