# Supplementary material for: Allelic Variation in CXCL16 Determines CD3+ T Lymphocyte Susceptibility to Equine Arteritis Virus Infection and Establishment of Long-Term Carrier State in the Stallion
Source: PLoS Genet. 2016 Dec 8;12(12):e1006467. doi: 10.1371/journal.pgen.1006467 (PMC5145142; doi:10.1371/journal.pgen.1006467)
Supplement: S1 Table — (DOCX) [file pgen.1006467.s002.docx]

**S1 Table. Ensembl and NCBI gene identification numbers.**

| rs Number | Genomic Coordinates | Gene^1^ | SNP^2^ | CXCL16  XM_  001504756^3^ | Amino acid residue^2^ | CXCL16  XP_  001504806^3^ |  |  |
| --- | --- | --- | --- | --- | --- | --- | --- | --- |
| rs395376880 | 48640104 | WSCD1 | ENSCAP00000005826:c.367T🡪C |  | ENSCAP00000005826Trp123Arg |  |  |  |
| ss1973464442 | 49070071 | NLRP1 | ENSCAP00000006832:c.631T🡪C |  | ENSCAP00000006832Ser211Pro |  |  |  |
| rs68886110 | 49084976 | NLRP1 | ENSCAP00000006832:c.[2349-1271T🡪C];  ENSCAP00000007007:c.[1585T🡪C] |  | ENSCAP00000007007:p.Trp529Arg |  |  |  |
| rs395626725 | 49427602 | ZNF (1425) | ENSCAP00000014525:c.[338A🡪G];  ENSCAP00000014599:c.[335A🡪G];  ENSCAP00000014694:c.[311A🡪G] |  | ENSCAP00000014525:p.[Glu113Gly]  ENSCAP00000014599:p.[Glu112Gly]  ENSCAP00000014694:p.[Glu104Gly] |  |  |  |
| rs396124728 | 49438260 | ZNF (14599) | ENSCAP00000014599:c.1480A🡪G |  | ENSCAP00000014599:p.Ile494Val |  |  |  |
| rs394122974 | 49457063 | ZNF (1660) | ENSCAP0000001660:c.184A🡪G |  | ENSCAP0000001660:p.Met62Val |  |  |  |
| rs782829411 | 49746951 | CXCL16 | ENSCAP00000015940:c.53A🡪T | c.715A🡪T | ENSCAP00000015940:p.Tyr18Phe | p.Tyr40Phe |  |  |
| ss1973464526 | 49746977 | CXCL16 | ENSCAP00000015940:c.79G🡪C | c. 801G🡪C | ENSCAP00000015940:p.Asp27His | p.Asp49His |  |  |
| rs782894239 | 49746980 | CXCL16 | ENSCAP00000015940:c.82T🡪A | c.804T🡪A | ENSCAP00000015940:p.Phe28Ile | p.Phe50Ile |  |  |
| rs782838921 | 49746986 | CXCL16 | ENSCAP00000015940:c.88G🡪A | c.810G🡪A | ENSCAP00000015940:Glu30Lys | p.Glu52Lys |  |  |
| ss1973464541 | 50591820 | SHBG | ENSCAP00000023073:c.1082C🡪A |  | ENSCAP00000023073:p.Pro361Gln |  |  |  |
| rs68875925 | 50838580 | KCNAB3 | ENSCAP00000009140;c.586A🡪G |  | ENSCAP00000009140Ile196Val |  |  |  |

*^1^WSCD1 - WSC Domain containing 1; NLRP1 - NLR family pyrin domain containing 1; ZNF - Zinc finger protein; CXCL16 - C-X-C motif chemokine ligand 16; SHBG - sex hormone binding globulin; KCNAB3 - potassium voltage-gated channel, shaker-related subfamily, beta member 3.*

*^2^ENSEMBL reference sequences*

*^3^NCBI reference sequences*
