# Supplementary material for: Allelic Variation in CXCL16 Determines CD3+ T Lymphocyte Susceptibility to Equine Arteritis Virus Infection and Establishment of Long-Term Carrier State in the Stallion
Source: PLoS Genet. 2016 Dec 8;12(12):e1006467. doi: 10.1371/journal.pgen.1006467 (PMC5145142; doi:10.1371/journal.pgen.1006467)
Supplement: S2 Table — (DOCX) [file pgen.1006467.s003.docx]

**S2 Table. Primers used in PCR amplification and sequencing of equine genes located in ECA11.**

| SNP Genomic Coordinates ECA11 | rs Number | Gene | Forward Primer Sequence (5′ to 3′) | Genomic Coordinates | Reverse Primer Sequence (5′ to 3′) | Genomic Coordinates ECA11 | Product Size |
| --- | --- | --- | --- | --- | --- | --- | --- |
| 48640104 | rs 395376880 | WSCD1 | CAGCTCCTACGTCTACGCTG | 11:48640272-48640291 | CAGCTTCTCTCTCCTGCAGG | 11:48639878-48639897 | 394 bp |
| 49070071 | ss1973464442 | NLRP1 | ACCCTGATTCCCTTTCCAAG | 11:49069893-49069912 | CACACCTTTGCCTCACTGAG | 11:49070122-49070138 | 245 bp |
| 49084976 | rs 68886110 | NLRP1 | CTGCTCAGACATGCCCTGG | 11:49084870-49084888 | GACGAGTAGTGTAGGTCTGTCC | 11:49085048-49085069 | 199 bp |
| 49427602 | rs 395626725 | ZNF (1425) | CAGAGCTCTAATCTGGTAAGACAT | 11:49427475-49427498 | GGTTTCTCCCCACTGTGGAG | 11:49427841-49427860 | 385 bp |
| 49438260 | rs 396124728 | ZNF (14599) | GAAGAACCTTCCAGGGCAGC | 11:49438153-49438172 | GCTCCATCTGAACGCTTTTCC | 11:49438477-49438497 | 344 bp |
| 49457063 | rs 394122974 | ZNF (1660) | CTGAGCCACATGGGGCAATA | 11:49457175-49457194 | GAAGTTTTGGCCAGACGCAG | 11:49456878-49456897 | 316 bp |
| 49746951 | rs 782829411 | CXCL16 | CGGTGGGTTGGAGGCTAA | 11:49746821-49746838 | GACCAGAGAGGGTCCCAGA | 11:49747083-49747101 | 280 bp |
| 49746977 | ss1973464526 | CXCL16 | " | " | " | " | " |
| 49746980 | rs 782894239 | CXCL16 | " | " | " | " | " |
| 49746986 | rs 782838921 | CXCL16 | " | " | " | " | " |
| 50591820 | ss1973464541 | SHBG | CTAATGCCCCCTCCATCG | 11:50591744-50591761 | GGGGTTCTTAGGTGGGGATT | 11:50591910-50591929 | 185 bp |
| 50838580 | rs 68875925 | KCNAB3 | CCTGAAGTGGCTGGGTATGGG | 11:50838776-50838795 | CCTGTGGAACTTAGGAGGCC | 11:50838463-50838482 | 313 bp |
| 49084976 | rs 68886110 | NLRP1 | GCTGTCGGCAATGATCGTTT | 11:49084892-49084911 | GTCTCTGACACAGCATGGCT | 11:49085070-49085089 | 197 bp |
| 49084976 | rs 68886110 | NLRP1 | AAGCATTTGTACTTCTGGAGCTG | 11:49084926-49084948 | AAACCTGCAAACCTGGGCTGCC | 11:49085016-49085038 | 112 bp |
